# The role of aromatase inhibitors in ameliorating deleterious effects of ovarian stimulation on outcome of infertility treatment

**DOI:** 10.1186/1477-7827-3-54

**Published:** 2005-10-04

**Authors:** Mohamed FM Mitwally, Robert F Casper, Michael P Diamond

**Affiliations:** 1 Division of Reproductive Endocrinology & Infertility, Department of Obstetrics & Gynecology, Wayne State University, Detroit, Michigan, USA; 2 Reproductive Sciences Division, Department of Obstetrics & Gynecology, University of Toronto, Toronto, Ontario, Canada

## Abstract

Clinical utilization of ovulation stimulation to facilitate the ability of a couple to conceive has not only provided a valuable therapeutic approach, but has also yielded extensive information on the physiology of ovarian follicular recruitment, endometrial receptivity and early embryo competency. One of the consequences of the use of fertility enhancing agents for ovarian stimulation has been the creation of a hyperestrogenic state, which may influence each of these parameters. Use of aromatase inhibitors reduces hyperestrogenism inevitably attained during ovarian stimulation. In addition, the adjunct use of aromatase inhibitors during ovarian stimulation reduces amount of gonadotropins required for optimum stimulation. The unique approach of reducing hyperestrogenism, as well as lowering amount of gonadotropins without affecting the number of mature ovarian follicles is an exciting strategy that could result in improvement in the treatment outcome by ameliorating the deleterious effects of the ovarian stimulation on follicular development, endometrial receptivity, as well as oocyte and embryo quality.

## PART ONE

### 1 Introduction

Current epidemiological evidence suggests that 15% of couples will experience infertility. Background prevalence rates now appear to be reasonably stable, but there is evidence of an increase in the rate of referrals for medical help [1,2]. Farley and Belsey, 1988 [3], have reported estimates of the prevalence (percentage) of primary infertility by region and country. They estimated 6% for North America, 5.4% for Europe, 3% for the Middle East, 10.1% for Africa, 4.8% for Asia and Oceania, 3.1% for Latin America and 6.5% for the Caribbean. The American Society for Reproductive Medicine (ASRM) estimates that 5 million American heterosexual couples report difficulties in achieving a viable pregnancy, of which 1.3 million seek advice for the problem [4].

### 2 Ovarian stimulation and assisted reproduction for infertility management

After correcting the abnormalities detected during the diagnostic workup, ovulation induction is usually performed either for treatment of anovulation/oligo-ovulation, or empirically in regularly ovulating women. This approach results in a pregnancy rate of around 8%–15% per cycle depending on the agents used for ovulation induction and the characteristics of the couple, such as the woman's age and the presence or absence of a male factor. Couples who do not become pregnant with ovulation induction alone then undergo more sophisticated treatment modalities including intrauterine insemination (IUI) and in-vitro fertilization and embryo transfer (IVF-ET) as a treatment of last resort [5].

Since the birth of Louise Brown in 1978, IVF-ET has become the therapeutic mainstay for female infertility. It has become generally accepted as therapy for a wide array of fertility problems, and has been accompanied by the rapid expansion of IVF-ET clinics worldwide resulting in more than 1% of babies being conceived by IVF-ET in western countries [6].

#### 2.1 Ovarian stimulation for assisted reproduction

In most assisted reproduction programs, gonadotropins are used alone or in combination to stimulate the growth and maturation of multiple follicles. This is essential because of the need to recruit a greater number of follicles, which provides the opportunity for retrieval of a large number of oocytes. This would improve the chance for fertilization of multiple oocytes and thereby allow an increased number of embryos for transfer in order to give acceptable success rates. Recent advances in the understanding of ovarian stimulation, the techniques of oocyte retrieval, the handling of gametes, the methods of assisted fertilization and improved conditions of culture media have steadily increased the fertilization rate. Fertilization rates of 60–70% can now be expected when conventional insemination, or even higher when intracytoplasmic sperm injection (ICSI) are carried out. However, there has not been a corresponding increase in implantation rates, which have remained steady at overall rates around 10%–15% [6].

#### 2.2 Low implantation rates with assisted reproduction

Throughout the last five decades, a progressive series of revolutionary techniques have been developed to overcome infertility, starting with the successful fertilization of human oocytes in vitro [7] and followed nearly 10 years later by the birth of the first IVF-ET baby [8]. Several other new developments in assisted reproduction have emerged, including cryopreservation and storage of embryos for later transfer [9], fertilization of oocytes with a single injected spermatozoon to alleviate severe male infertility i.e. ICSI [10] and diagnosis of genetic defects from preimplantation embryos prior to intrauterine transfer [11]. However, although IVF-ET is now a standard, well-established treatment for infertility, success rates remain relatively low, with only about 33% of cycles resulting in pregnancy [12]. This is believed to be due to the low implantation rate that has not significantly increased as fertilization rates [13]. Efforts are being made to improve implantation rates after IVF-ET by improving culture conditions, optimizing gamete quality and developing new techniques of selecting viable embryos for transfer without significant success. For this reason, multiple embryos are generally transferred to improve pregnancy rates, but this has resulted in an unacceptably high rate of multiple-gestation pregnancies [14].

Although governed by multiple interactive events, embryo implantation depends mainly on the quality of embryos and the status of uterine receptivity. During the last two decades, several developments in controlled ovarian hyperstimulation [COH], fertilization, and embryo culture techniques have led to an optimization in the number and quality of embryos available for ET. In contrast, uterine receptivity has failed to benefit from parallel improvements, and its disarrangement is likely to represent an important cause of the sub-optimal embryo implantation rates observed in IVF-ET [15].

#### 2.3 Poor outcome of infertility treatment associated with ovarian stimulation

In the following section we review in brief both animal and human evidence for the unfavorable outcome including impaired implantation and increased adverse outcomes in pregnancies achieved following ovarian stimulation when compared with spontaneous pregnancies.

##### 2.3.1 Animal data

Increased pre- and post-implantation embryonic loss has been reported in mammals [16-21] including rats [16,18], mice [17-19], murine [20] and hamsters [21], in association with ovarian hyperstimulation. These effects have been attributed to ovarian stimulation using standard doses of gonadotropins. At higher doses of gonadotropins, studies have found increased frequencies of oocyte aneuploidy, embryo mortality, fetal growth retardation and congenital abnormalities [22,23].

The poor outcome after ovarian stimulation has been attributed to adverse effects on the maternal side and or the gametes and embryo side. On the maternal side: inadequate uterine synchrony or receptivity has been reported. On the gamete and embryo side, ovarian stimulation has been found associated with chromosomal defects in the oocyte leading to increased lethality during the preimplantation stages [22,23]. However, because species-specific variations in implantation strategies exist, these differences preclude the formulation of a unifying theme for the molecular basis of this event.

##### 2.3.2 Human data

Many studies have found higher pregnancy rates in donor oocyte recipients than patients undergoing standard IVF-ET [24,25]. The higher success rates could be attributed to better quality oocytes from younger donors. However, in centers using a shared oocyte system, where the donor keeps half of the oocytes for herself, significantly higher pregnancy and implantation rates were found in the recipients [26].

Another evidence for adverse effects of ovarian stimulation on implantation is the higher implantation rate associated with IVF-ET in the natural or un-stimulated cycle. Although most studies were associated with a rather high proportion of cancelled cycles [25–75%] and a low clinical pregnancy rate per started cycle [range 0–23%], higher implantation rates have been reported (up to 30%) [27].

###### Adverse Obstetrical Outcome after ovarian stimulation

Induction of ovulation has been shown to raise the risk of miscarriage when compared with spontaneous pregnancies [28]. This was true even after controlling for advanced age, a known significant risk factor for miscarriage. Higher risks as compared with natural pregnancies are reported in pregnancies after IVF-ET, primarily owing to growth retardation and pre-term birth. Although this can be explained by the high multiple pregnancy rates in IVF-ET pregnancies [29], an increased rate of small for gestational age and pre-term birth children is reported in singleton IVF-ET pregnancies as compared with natural singleton pregnancies after adjustment for potential biases [30-32]. Moreover, twin pregnancies after assisted reproduction have a higher rate of perinatal mortality and lower birth weight as result of a higher rate of premature parturition when compared to spontaneously occurring twins [33,34]. In addition, it was reported that women who conceived multiple gestations through assisted reproductive technologies have a 2.1-fold higher risk of preeclampsia than those who conceive spontaneously [35]. Pregnancies associated with severe ovarian hyperstimulation syndrome have been found to be complicated with increased miscarriage rates [36,37]

While ovarian stimulation has been suggested to contribute, at least in part, for the adverse obstetric outcome other studies have shown that infertility itself is a factor that leads to increased obstetric risks and that sub-fertility is a predictor for low birth weight. Some believe that it is the cause of infertility itself, rather than the use of ovarian stimulation, that is the reason behind poor obstetric outcome after infertility treatment [38,39].

### 3 Postulated mechanisms behind adverse effects of ovarian stimulation

Several causes and targets have been suggested to explain the poor outcome associated with ovarian stimulation (Table 1). These include (1) supraphysiological levels of estrogen, and other steroids and peptides, attained during ovarian hyperstimulation, (2) the use of exogenous gonadotropins as well as other medications applied during ovarian stimulation such as Gonadotropin releasing hormone (GnRH) analogues (agonists and antagonists), human chorionic gonadotropin (hCG) and clomiphene citrate (3) Other possible undetermined factors. These factors are believed to act through their effects on (i) the endometrium, (ii) the developing oocyte, (iii) the developing embryo, (iv) the ovaries and corpus luteum, (v) the pituitary gland, and (vi) possibly on other targets such as fallopian tubes, the coagulation system, as well as the early developing placenta.

**Table 1:** Postulated mechanisms behind less favorable treatment outcome (reduced implantation rate and increased adverse obstetric outcome) after ovarian hyperstimulation

Causes:	Targets
1-Supraphysiological estrogen levels attained during ovarian stimulation 2-Medications used during ovarian stimulation: •Clomiphene citrate •Gonadotropins •GnRH analogues: agonists & antagonists •HCG used to trigger ovulation 3-Other probable causes: abnormal levels of other hormones, peptides and local autocrine mediators associated with ovarian stimulation	1-Endometrium 2-Developing oocyte 3-Sperm 4-Developing embryo 5-Maternal endocrine system: ovaries (corpus luteum), pituitary gland and hypothalamus. 6-Other probable targets e.g. •Fallopian tubes •Early developing placenta •Leptin-mediated effects •Coagulation system

#### 3.1 Effect of supraphysiological levels of estrogen

It is believed that the supraphysiological levels of estrogen, attained during ovarian stimulation, may explain, at least in part, for the adverse effects of ovarian stimulation on the outcome of infertility treatment [40-42]. Different mechanisms have been suggested for the deleterious effects of the supraphysiological levels of estrogen attained during ovarian stimulation Table 2.

**Table 2:** Deleterious effects of the supraphysiological estrogen levels attained during ovarian stimulation:

1-Effect on the endometrium: most of the available evidence
a: Dys-synchronization of the implantation window.
b: Abnormal Temporal expression of the endometrial pinopodes.
c: Defective endometrial estrogen and progesterone receptors.
d: Impaired endometrial blood flow
e: Abnormal endometrial integrins expression
2-Effect on the developing oocyte
a: Effect on chromosomal and cytogenetic integrity of the oocyte
b: Effect on the mitochondrial function
3-Effect on the sperm causing possible premature acrosome reaction and deactivation
4-Effect on the developing embryo and blastocyst hatching
5-Effect on the ovaries and pituitary (defective corpus luteum function and luteal phase):
a: Defective LH secretion:
- Abnormal LH surge
- Abnormal LH tonic pulse
b: Defective corpus luteum function
6-Other probable targets:
I-Leptin
II-Coagulation system: III-Fallopian tubes
• a: Effect on the ductal environment and ductal fluid
• b: Effect on the ductal transfer and motility
IV-Early developing placenta

Considering all the patients together, significant decreases in pregnancy and implantation rates were observed when estradiol (E_2_) concentrations were > 2500 pg/ml [>9000 pmol/L.] compared with patients having lower E_2 _concentrations. High serum E_2 _concentrations on the day of hCG injection in high and normal responder patients, regardless of the number of oocytes retrieved and the serum progesterone concentration were found to be detrimental to uterine receptivity [42]. Later, it has been shown that a significant reduction in the implantation and pregnancy rates occurred in almost all women with a higher serum E_2 _concentration of ~5–6000 pg/ml [19–22,000 pmol/L] [43,44]. Recently, we have presented data showing that high E_2 _levels are associated with less favorable treatment outcome in women undergoing controlled ovarian hyperstimulation and IVF-ET [45-51]. We studied the effect of E_2 _by looking at the area under the curve (AUC) for E_2 _levels along the stimulation cycle [45-51]. We believe this is more accurate than looking at a single measurement of E_2 _e.g. one day of hCG administration [46]. In addition to studying the AUC for E_2 _levels [45,46], we looked at the E2 production per mature follicle and per gonadotropin dose administered during COH [48] and the effect of age on these parameters [47]. We found significant correlation between these parameters and the outcome of IVF-ET treatment showing that high E2 levels are associated with lower clinical pregnancy and implantation rates [45-49] in addition to increased adverse obstetric outcomes including higher miscarriage rate [49] and lower birth weight [50]. Successful IVF-ET treatment cycles were associated with lower AUC-E2 compared to unsuccessful cycles at the same patients [49].

##### 3.1.1 Effect of supraphysiological estrogen levels on the endometrium

One of the causes for the reduced implantation rates associated with ovarian stimulation may be an impairment of endometrial receptivity, due to high concentrations of sex steroids. This suggestion is supported by higher implantation rates in hormonal replacement treatment cycles after ovum donation, as opposed to the standard IVF-ET cycles as explained earlier.

###### Effect on Implantation Window

It is generally believed that the embryo-uterine interactions leading to implantation can only succeed when embryonic development is synchronized with the preparation of the endometrium to the receptive state. Typically, this means that the embryos have reached the blastocyst stage and that the endometrium has undergone certain hormone-dependent changes during a specific time window in the preimplantation phase that prepare it to be receptive to the developing blastocyst [52].

The concept of endometrial receptivity introduced by Psychoyos [53,54], has been shown to last only for several hours, thereby determining a narrow nidation window. The concept of an "implantation window" or "receptive endometrium" was initially established in rodents. In the rat, the fertilized embryo reaches the uterus on day 4 after fertilization, and implantation occurs in the afternoon of day 5 [53,54]. In humans, the ovum is fertilized in the fallopian tube, arrives in the uterine cavity around day 17 (day 14 is taken as day of ovulation of a 28-day cycle), and remains there as a free-floating embryo until about day 19; implantation then occurs between days 19 to 22 [55,56]. However, the precise timing and molecular basis of the receptive window in the human remain undefined. Unfortunately, endometrial receptivity knowledge in the human is limited due to the obvious experimental drawbacks and the lack of specific criteria to define a receptive endometrium.

In the literature, there is controversy regarding the effect of ovarian stimulation on endometrial development. Most of the investigators have reported adverse effects of high estrogen levels on endometrial development but there was no consensus on the actual effect. Some have shown endometrial advancement [57-61] while others showed endometrial retardation [62,63]. However, all studies confirm direct deleterious effects on the endometrial development that jeopardize the chance of implantation due to the lack of synchronization between the development of the endometrium and the early embryo development. Implantation failure has been suggested to result from the disparity in maturation between the endometrial stroma and the epithelium observed in histology. Since a paracrine communication between the epithelium and the stroma may be important at the beginning of implantation, this disparity could compromise uterine receptivity or early trophoblastic invasion [64,65].

The effect of high E_2 _concentrations (> 20,000 pmol/L) was found to be associated with gland-stromal dys-synchrony, which indicated a deficient secretory transformation of the endometrium that represents a sub-optimal endometrial environment for implantation. This finding substantiates clinical observation of significantly lower pregnancy rates in IVF-ET cycles of women with high E_2 _concentrations. In these patients, there was a marked stromal edema associated with a significantly greater number of stromal vessels, which suggested advanced stromal maturation [44].

In these studies, definitions of supraphysiological hormonal concentrations were variable. Also, the timing of the biopsies and the drug regimens used for ovarian stimulation were different. This, in addition to the variation in endometrial response to different E_2 _concentrations, may explain the disagreements reported in the literature [66]. Moreover, both the premature progesterone serum elevation, which occurs in 30% of stimulated IVF-ET cycles before hCG administration, and the advanced post-ovulatory rise in progesterone serum concentrations are believed to be responsible for the advanced endometrial development associated with ovarian stimulation [67,68]. However, other studies failed to confirm these observations [69,70].

###### Effect on endometrial pinopodes

At the time of implantation, the apical membranes of the epithelial cells lining the uterine cavity develop large and smooth membrane projections, named pinocytes due to their pinocytotic function [71]. Their development is progesterone-dependent with strict correlation with the implantation window in the rodents [72]. Similar structures have been seen in the human endometrium [73]. The number of pinopodes was found to have a strong correlation with implantation after embryo transfer [74,75].

Hormonal treatment has been shown to be associated with changes in the timing of pinopode formation. During ovarian stimulation with clomiphene citrate followed by human menopausal gonadotropins (hMG)/hCG, fully developed pinopodes were found 2 to 3 days earlier [76]. In contrast, with E_2 _and progesterone treatment, fully developed pinopodes were found to be about two days later. Pinopodes were found to form as early as 4 days after hCG administration [59].

Nikas et al. [61], studied the temporal expression of pinopodes as a specific marker for receptivity in IVF-ET cycles induced by gonadotropins, compared to women with regular menstrual periods and proven fertility who served as controls. They did not find ovarian stimulation to affect endometrial pinopode formation in terms of quantity and life span. Instead, the cycle days when pinopodes formed were specific to the individual, being on average 1–2 days earlier in cycles with ovarian stimulation than in natural cycles. These changes in pinopode expression may reflect shifts in the window of receptivity, resulting in ovo-endometrial asynchrony and limiting implantation success in IVF-ET [61]. In IVF-ET, embryonic development is probably delayed while the uterus is advanced, resulting in an early closure of the nidation window, before the embryo eventually reaches a stage capable of initiating implantation. These findings support the theory that reduced implantation rates in IVF-ET cycles could result from impaired or premature endometrial maturation.

###### Effect on estrogen and progesterone receptors in the endometrium

A lack of estrogen receptors (ER) has been reported [77] during ovarian stimulation cycles that rendered the endometrium functionally hypoestrogenic or hypoprogestogenic. Also, it has been reported that the expression of progesterone receptors (PR) in the endometrium was decreased in the early part of luteal phase after ovarian stimulation [78]. This premature PR decrease was consistent with an early high progesterone level [79]. Papanikolaou et al. [80], investigated prospectively the effect of multi-follicular ovarian stimulation for IVF on the late follicular phase endometrium histology and the expression of ER and PR. Endometrial biopsies were taken in a natural cycle on the day of the onset of the surge of the LH, and in a subsequent stimulation cycle on the day of hCG administration for final oocyte maturation. Histological examination of biopsies both in natural and stimulated cycles showed no secretory changes. However, in stimulated cycles, PR expression was significantly up-regulated compared to natural cycles in both glands (1.67 versus 1.34, P < 0.05) and stroma (1.98 versus 1.62, P < 0.05), whereas ER was down-regulated in glands (1.15 versus 1.43, P < 0.05). In IVF cycles, the progesterone measurements, although within normal values (range 0.8–1.4 microg/l), were significantly higher than in natural cycles (0.99 vs 0.63 microg/l, respectively, P = 0.008). An ongoing pregnancy rate of 37.5% was achieved in the stimulated cycles. The authors concluded that although the current study found no early secretory transformation in stimulated endometria before hCG administration, the ER and PR expression in these endometria was similar to the one described during the first days of the luteal phase in natural cycles. Supraphysiological concentrations of estradiol and subtle progesterone rises in the late follicular phase might be responsible for this modulated steroid receptor profile. They added that this phenomenon indicated accentuated maturation of the endometrium in IVF cycles from the pre-ovulatory phase onwards. [80].

Ovarian stimulation with GnRH-agonist/hMG was found to induce precocious secretory endometrial transformation around the time of oocyte retrieval. Compared to natural cycles, there was an imbalance between endometrial steroid receptor content, proliferation index, and maturation in the peri- and postovulatory phases of stimulated cycles. The lower ER in stimulated cycles on the day of oocyte pick up as compared to the natural cycle controls on the day of ovulation was mainly observed in the stroma, but failed to reach a significant difference in the glands. As for PR, the staining intensity of the stromal ER in the stimulated cycles was higher than that of luteal phase day 2 of a natural cycle. These findings suggest a relative imbalance in ER and PR content of the endometrium in stimulated cycles compared to their natural cycle counterparts. [79].

In conclusion, excessive ovarian response was suggested to lead to insufficient secretory transformation of the endometrium, as well as discordant glandular and stromal development at a time that coincides with the period of maximum uterine receptivity.

###### Effect on endometrial vasculature and endometrial blood flow

Angiogenesis has a critical role in female reproductive physiology. Growth of the endometrium and placentation is also accompanied by extensive angiogenesis. Thus, an actively maintained blood supply is an essential requirement for reproductive functions, including normal implantation [81]. Endometrial vasculature has been shown to play a prominent role in the early endometrial response to the implanting blastocyst, and vascular changes may contribute to uterine receptivity [82].

The introduction of transvaginal Doppler ultrasound makes the measurement of uterine artery blood flow possible, and at one time it was hoped that uterine arterial resistance changes might reflect uterine receptivity [83]. Applebaum first introduced the concept of evaluating uterine receptivity by a uterine score including the endometrial blood flow [84]. Different studies have demonstrated significant changes in the Doppler indices of uterine and ovarian vessels during ovarian stimulation and spontaneous cycles [85,86]. Basir et al., found evidence of impaired endometrial blood flow in association with significantly high estrogen levels in high responders to ovarian stimulation [87].

Although pregnancy outcome tended to be poor in patients with higher mean uterine arterial impedance indices, the predictive value of using a specific resistance index (RI) or pulsatility index [PI] variable in assessing endometrial receptivity seems to be limited [88]. One of the explanations is that the major uterine compartment is the myometrium and not the endometrium, and thus most of the blood passing through the uterine arteries never reaches the endometrium. A more logical approach would be to evaluate the vascularization around the endometrium directly in an attempt to assess endometrial receptivity.

Histological studies have confirmed that the sub-endometrial halo surrounding the endometrium represents the innermost layer of the myometrium, and compared with the outer myometrium, it consists of a distinct compartment of more tightly packed muscle cells with increased vascularity. Studies have shown that interactions between the junctional zone and the endometrium may play an important role in the implantation process [89,90] and endometrial-sub-endometrial blood flow distribution pattern assessed by transvaginal color Doppler before ET was found to correlate with the implantation and pregnancy rate after IVF-ET [91]. With the absence of sub-endometrial blood flow, even in the presence of other favorable parameters, no conception was achieved. By using a similar approach, Salle et al., calculated a uterine score in the secretory phase of the menstrual cycle preceding IVF-ET [92]. Immunocytochemistry study revealed that the sub-endometrial myometrium, also called the junctional zone myometrium or archimyometrium, exhibits a cyclic pattern of estrogen and PR expression that parallels that of the endometrium [93]. Moreover, the responsiveness of the junctional zone has been shown to be associated with implantation success during IVF-ET treatment [90].

Many investigators have also noted the correlation of junctional zone contractions with pregnancy outcome in both natural [94] and assisted reproduction cycles [95]. Less-active junctional zone contractility is associated with higher pregnancy rates

Kupesic et al. compared the 2-D and 3-D ultrasonographic scoring systems by combining parameters including endometrial thickness, volume, echogenicity, and sub-endometrial blood flow. They found the two systems had similar efficiencies in predicting pregnancy outcome of IVF-ET procedures [96]. Several investigators noticed that when the endometrial and sub-endometrial flow parameters were combined, significant differences were found between pregnant and nonpregnant patients [97-99]. In contrast, there was no significant difference if attention was only focused on intraendometrial or sub-endometrial blood flow [100]. These results imply that the endometrial/sub-endometrial area must be considered as a whole in evaluating endometrial perfusion.

One hallmark of implantation is increased vascular permeability at the implantation site. Vasoactive agents, including histamine, platelet-activating factor, vascular endothelial growth factor, and eicosanoids, have been studied during implantation [101,102]. Vaginal E_2 _administration improves endometrial proliferation and uterine perfusion, presumably because of combined local and systemic effects, but may interfere with P-induced uterine relaxation [103].

In a study by Basir et al., [104] the investigators compared the hemodynamic parameters of the utero-ovarian vasculature and the endometrial spiral arteries of women who showed a moderate response with women whose E_2 _concentrations were in excess of 20,000 pmol/l after ovarian stimulation. Despite low uterine PI and RI, the endometrial blood flow in high responders appears to be impaired. The authors concluded that this might contribute to the decline in implantation efficiency noted in high responders. The decreased endometrial blood flow despite the increased blood flow in the uterine arteries may indicate a shunt of blood flow from the endometrium into the myometrium.

The authors [104] found low pulsatility index and resistance index of the ovarian arteries indicating neovascularization and increased capillary permeability in the ovarian tissue of high responders. The authors suggested that the blood flow might be directed through the utero-ovarian collaterals to the ovaries. However, because the sample size in this study was small (19 patients with E_2 _> 20,000 pmol/L), further larger prospective studies are required to confirm the effect of excessively high concentrations of serum E_2 _on endometrial blood flow.

Moreover, the increase in hormonal concentrations in the peripheral plasma leads to a decrease in peripheral vascular resistance [105] and a decreased contractility of the uterine muscles. This results in relaxation and opening up of the small uterine vascular channels, which may also cause an increase in the capillary permeability. In a study on the endometrial morphological changes at high concentrations of E_2_, a significantly greater number of vessels and endometrial edema in women who responded excessively to ovarian stimulation was demonstrated. Therefore, it was postulated that the blood flow through these minute endometrial vessels may be very slow and the weak Doppler flow signals arising from them could not be picked up by the color Doppler despite low uterine PI and RI. The increase in capillary permeability and dilatation leads to extravasation of fluid from the intercellular to extracellular compartments, and hence endometrial edema [106]. In another study the investigators suggested that the blood flow per capillary might actually be reduced during edema [107].

Successful implantation and continuing development of implanted embryo depends on a complex series of cellular and molecular events between the blastocyst and the endometrium [108]. The decline in blood flow could therefore impede the exchange of essential nutrients, bioactive molecules and reactive compounds that are vital for implantation with absent endometrial and intra-endometrial vascularization appeared to be a useful predictor of failure of implantation in IVF-ET cycles [109].

Whether fertilization occurs in vivo or in vitro, most human embryos will not develop through gestation [110] with a very high proportion of developmental failure during the preimplantation stages associated with chromosomal defects of oocyte origin [111]. Van Blerkom suggested that the dissolved oxygen content of pre-ovulatory follicular fluid and the developmental competence of the corresponding oocyte were related [112]. A developmentally significant association between the chromosomal normality of the human oocyte and the level of intra-follicular oxygen and peri-follicular vascularity was reported suggesting that hypoxic intra-follicular conditions that result from the failure of an appropriate microvasculature to develop around the growing or pre-ovulatory follicle(s) could be a proximate cause of the maternal-age-related increase in the incidence of trisomic conditions [112-114].

###### Effect on Integrins

Integrins are a family of cell adhesion molecules. Previous work has shown the expression of integrins in the endometrium changes during the menstrual cycle [115]. Three integrins in particular (_1_β_1_, _4_β_1 _and vβ_3_) are thought to play a vital role in implantation as all are expressed during the 'implantation window'. The β_3 _and β_1 _integrins have also been shown to be reduced in infertile patients using flow cytometry [116]. Aberrant patterns of integrin expression have also been associated with certain diagnoses in infertile patients, including luteal phase defects, endometriosis, hydrosalpinx and unexplained infertility [117].

The exact role of integrins remains controversial and results have not been duplicated in all studies. Creus and co-workers showed no difference in integrin expression in patients who became spontaneously pregnant compared with those that do not [118]. Thomas et al., demonstrated that integrin expression seems to be reduced in the glandular epithelium in the endometrium after ovulation induction, irrespective of the dating. The authors concluded that there might be an ideal E_2 _level that should be reached during IVF-ET treatment as low estrogen levels might reduce the yield of oocytes, but high levels might impair the receptivity of the endometrium reducing integrin expression and leading to lower implantation rates [119].

##### 3.1.2 Effect of supraphysiological estrogen levels on the developing oocyte (follicular and oocyte development)

As the contributor of the bulk of the cytoplasm to the zygote and half of its nuclear DNA, the key importance of the oocyte is unquestionable. The principle, that embryogenesis is rooted in oogenesis, has been understood for nearly a century, and is now becoming better understood at the molecular level. During follicular growth, the oocyte is in close contact with granulosa cells through gap junctions and is therefore under the influence of the follicular environment. Since oocyte maturation is such a long process, any adverse events occurring during this period can damage vital structural molecules or whole organelles, resulting in oocytes with reduced developmental competence. These oocytes may in turn give rise to preimplantation embryos with a compromised viability, and hence, a reduced implantation potential [120]. The ER gene is expressed in the human cumulus-oocyte complexes and oocytes but not in granulosa/cumulus cells which might suggest a lack of receptor-mediated autocrine effect of estrogen during folliculogenesis. Conversely, estrogen secreted by granulosa/cumulus cells, may exert a paracrine effect to influence oocyte maturation and fertilization competence directly [121]. It was reported that retrieval of >10 oocytes during IVF-ET cycles was correlated with oocytes of lower quality, as manifested by a decrease in the fertilization rate [122].

###### Effect on the chromosomal structure and cytogenetics of the oocytes

Studies with animal systems have indicated that a single cycle of ovarian stimulation can have adverse effects on oocyte competence during early development, and may well have downstream effects on the normality of fetal growth and development. Whether developmental defects could result from chromosomal malsegregation during ovarian stimulation-induced meiotic maturation has been examined in the mouse system. Earlier studies compared oocytes obtained after spontaneous or hormonally induced ovulation and found no increase in the incidence of non-disjunction or oocyte aneuploidy after ovarian stimulation [123]. In contrast, cytogenetic analysis of pronuclear stage mouse eggs after single cycle of ovarian stimulation showed chromosomal aberrations largely confined to the female pronucleus, indicating developmental compromise prior to fertilization [124]. Sengoku and Dukelow found comparable frequencies of aneuploidy for cleavage-stage hamster embryos produced in natural and pregnant mare serum gonadotropins (PMSG)-stimulated cycles, indicating that peri-implantation mortality may have an epigenetic origin [125].

In IVF-ET programs, the high rate of oocyte recovery and successful fertilization in vitro contrasts with a relatively high rate of conception failures, as even the most advanced IVF-ET clinics can provide a 33% success rate per cycle at best [12], despite multiple embryo transfers. The possible reason for this conception failure is the high frequency of lethal chromosomal abnormalities that prevents embryonic development beyond the pre- and post-implantation stages. The high frequency of spontaneous abortions also indicates that the proportion of embryos with genetic defects is significant. Cytogenetic analysis of the early stages of cleavage indicates that chromosomal aberrations may be found in 35–44% of pre-implantation embryos produced in vitro [126]. It has to be emphasized that the majority of pre-implantation embryos carry numerical chromosomal defects. However, the high incidence of chromosomal anomalies is presumably biased by the fact that only embryos with a poor morphological score were analyzed (discarded embryos). It nevertheless indicates that the low implantation rates of human pre-implantation embryos in IVF-ET programs are the consequence of natural selection [127]. Aneuploidy is the most common abnormality found in normally developing embryos following ovarian stimulation and IVF-ET [128], but other aberrations were also revealed. Polyploidy and multinucleation were frequently described in arrested embryos

Estrogen is well known to induce chromosomal and cytogenetic damage. The natural hormone E_2 _has clearly been shown to induce alterations in chromosome number such as losses or gains of whole chromosomes [129,130], chromosome translocations [131,132], and gene amplifications [133,134]. In addition, there is preliminary evidence of estrogen-induced gene mutations and gene deletions [135,136].

The synthetic estrogen, diethylstilbestrol (DES) causes a severe yet reversible deterioration of meiotic spindle microtubule organization during maturation of the mouse oocytes [137]. High doses of E_2 _were found to induce numerical chromosome changes (both chromosome gains and losses) similar to the reported observations with the synthetic estrogen, diethylstilbestrol [129]. Estrogen is also known to cause direct DNA damage via its catecholestrogen metabolites [138,139]. There are several types of free radical-mediated DNA damage, which are induced by estrogens and/or their metabolites [140-142].

As explained earlier, supraphysiological estrogen levels associated with ovarian stimulation impair the uterovarian blood flow resulting in follicular hypoxia. Gaulden proposed that follicular hypoxia might have a potent adverse influence on spindle organization and the normality of chromosomal segregation in the human oocyte [143].

###### Effect on the mitochondrial function of the oocytes and embryos

Van Blerkom has suggested that the developmental competence of mouse and human early embryos is related to the metabolic capacity of the mitochondria. It is thought that mitochondrial replication does not begin until after implantation, and that paternal contribution is minimal. Therefore the preimplantation embryo is completely reliant on maternally inherited mitochondria in the oocyte. Deletions and mutations in oocyte mitochondrial DNA may lead to mitochondrial dysfunction, influencing energy production and apoptosis in oocytes and early embryos, resulting in aberrant chromosomal segregation or developmental arrest [144].

The classical model of E_2 _action has been described to be mediated by cytoplasmic/nuclear partitioning receptor proteins that stimulate gene transcription upon binding to specific DNA sequences [145]. However, there are increasing functional evidences for extra nuclear/cytoplasmic localization of steroid hormone receptors. Several studies showing rapid non-genomic actions of steroids have led to speculate about the existence of cell-surface resident receptor forms [146-148]. Reports have documented the presence of estrogen binding proteins localized at the plasma membrane [149,150]. Independently, the known direct effects of various steroids on mitochondrial gene transcription support the idea of receptor attachment to the mitochondrial genome [151]. It was also identified early that estrogen specific binding sites were associated with mitochondrial and microsomal structures [152].

The mitochondrial-enriched subfraction represented an important source of E_2 _binding, where the steroid was recognized in a stereospecific and high affinity manner. The existence of mitochondrial and membrane estrogen binding sites correlated with the presence of ER but mainly with ER proteins. Using macromolecular E_2 _derivatives in Ligand Blot studies, both mitochondrial and membrane estrogen binding proteins were found in the uterus, and the ovary. This differential cellular partitioning of ER and forms may contribute to the known diversity of E_2 _effects in target organs [153].

Recently, in myocardial cell model, it was reported that at physiological concentrations, which do not inhibit mitochondrial functions, estrogens can protect heart mitochondria from the loss of cytochrome c induced by high calcium, and this might be one of the possible mechanisms by which estrogens preserve myocardial cell viability after ischemia/reperfusion [154]. High concentrations of estrogens (50–100 M) have been found to have a damaging effect on mitochondrial functions by strongly inhibiting mitochondrial respiration and membrane potential presumably due to decreased activity of the respiratory chain. The inhibition of the respiratory chain may be due to non-specific binding of estrogens to hydrophobic regions of the mitochondrial membranes, which may change protein/lipid interactions, disturb electron transport through the inner mitochondrial membrane and reduce membrane potential [155].

We postulate that supraphysiological levels of estrogen attained during ovarian stimulation may affect the mitochondrial function of the developing oocyte and embryo. This could be one of the mechanisms behind impaired developmental capacity of the oocytes and embryos obtained after ovarian stimulation.

##### 3.1.3 Effect of supraphysiological estrogen levels on the spermatozoa and sperm/oocyte interaction

Sperm are exposed to estrogens within the male tract, and P450 aromatase has also been identified in human spermatozoa [156]. This raises the possibility that the spermatozoa can provide a continuing local source of estrogens in the epididymis, as well as while in the female tract on its journey to fertilize the oocyte. Inside the female genital tract, the spermatozoa would also be exposed to estrogens, particularly in tubal fluid following follicle rupture and when in close vicinity to released oocytes [157].

###### Estrogen receptors in the spermatozoa

Non-genomic effects of estrogens have also been reported in several cell types including the spermatozoa [158]. Immunohistochemical detection of ER has been reported for human spermatozoa, with distribution in both the head and flagellum [159].

###### Possible effect of estrogen on the spermatozoa function

After ejaculation, in vitro, the spermatozoa are initially unable to fertilize [160]. The spermatozoa acquire the capacity to fertilize, after a certain period of time that is species-specific when exposed to an appropriate environment either in vivo or in vitro. This process is called 'sperm capacitation' [161]. Capacitated spermatozoa are able to express hyperactivated motility, to undergo the acrosome reaction, and to fertilize an oocyte [160]. Estrogens are believed to have a possible effect on these events (capacitation and acrosome reaction), which would have important consequences on fertility in vivo.

Recently, Adeoya-Osiguwa provided evidence that E_2 _and environmental estrogens can significantly stimulate mammalian sperm capacitation, acrosome reactions. They found that in uncapacitated cells, E_2 _at 00.001 μmol/l, significantly stimulated capacitation and acrosome reactions while in capacitated cells, E_2 _had no effect. The authors concluded that whether these responses have effects on fertility in vivo remains to be determined, along with the mechanisms of action involved [157]. It is pertinent to mention here that the average E_2 _concentrations in follicular fluid from mature oocyte are in the micromolar range [162,163].

The regulation of capacitation is very important in mammalian fertilization as evidence suggests that once capacitation has been initiated it will usually continue unchecked, frequently resulting in the spermatozoa undergoing spontaneous acrosome reactions and thus becoming non-fertilizing [164].

Adeoya-Osiguwa argued that E_2 _and the environmental estrogens appear primarily to stimulate the spermatozoa, accelerating the rate of capacitation and then promoting 'over-capacitation' in at least some of the cells, resulting in the acrosome reaction [157]. Since already acrosome-reacted spermatozoa are non-fertilizing [160], similar responses occurring in vivo could reduce the number of potentially fertilizing cells and so have an undesirable effect on fertility. As capacitation and fertilization occur in the female reproductive tract, it is likely that any effects of environmental estrogens on sperm function would be more pronounced in the female, but effects on mature spermatozoa awaiting ejaculation cannot be ruled out.

##### 3.1.4 Effect of supraphysiological estrogen levels on the embryo

Mouse and human embryos, when cultured in vitro, undergo a delay in development compared with those grown in vivo. This delay can be caused by suboptimal culture conditions, but possible influences of ovarian stimulation cannot be excluded [165]. In order to determine if implantation failure associated with ovarian stimulation was due to abnormalities in the blastocysts or the endometrium, decidualization studies and embryo transfers to pseudopregnant recipients were performed [166,167]. The uterus of a large proportion of superovulated animals was unable to undergo decidualization in time, whereas embryo transfers to pseudopregnant females resulted in normally developing fetuses, which indicated that hormonally treated oocytes themselves were not affected. Some studies found that the E_2 _concentrations in the fresh cycle were not related to the success of frozen-thawed embryo transfer cycles indicating that embryo quality seemed unaffected by the high estrogen levels [168,169]. However, there is increasing evidence suggesting that ovarian stimulation and the associated high estrogen levels are detrimental on the embryo and associated with a decrease in the fertilization rate. When compared with blastocysts derived from naturally cycling mice, blastocysts that developed in vivo in superovulated mice were found to have fewer microvilli on their surface [170], a reduced [^35^S]-methionine uptake [171], and a lower cell number and mitotic index [172]. A reduced cell number and a two-fold decrease in viability post-transfer of embryos from gonadotropins-stimulated hamster females, was also observed [21]. Furthermore, it has been reported that the proportion of abnormal preimplantation embryos increases after superovulation, and that blastocysts have a smaller trophoblastic outgrowth in vitro [173]. Moreover, in mice as well as in humans, there is evidence for steroids being regulators of gene expression in the embryo and endometrium, and that embryo morphology and rate of development – both of which reflect embryo quality – have a genetic basis. Also, ovulation induction therapy has been found to be associated with an increased rate of mosaicism in the embryos, which fail to implant [52].

It has been proposed that high E_2 _levels after COH impair endometrial receptivity because oocyte quality, fertilization rate, and embryo cleavage (until day 2) were normal in patients with a high response [174] and the quality of embryos and the implantation rate seemed normal in subsequent frozen-thawed embryo transfer [43]. However, high E_2 _levels were found to be deleterious to embryo adhesion in vitro, mainly because they have a direct toxic effect on the embryo that may occur at the cleavage stage [175].

Mouse and human embryos, when cultured in vitro, undergo a delay in development compared with those grown in vivo. This delay can be caused by suboptimal culture conditions, but possible influences of ovarian stimulation cannot be excluded. In the mice, preimplantation embryonic development in vitro and in vivo was found to be negatively influenced by the ovarian stimulation itself, and results in an impaired blastocyst formation and fetal growth retardation at day 14 of gestation. The authors suggested that a similar negative effect of ovarian stimulation on oocyte and embryo quality seems likely in IVF-ET which might explain in part for the delay in embryonic development after IVF-ET, and for the low birth weight often observed after assisted reproductive technologies [165].

###### Effect of estrogen on blastocyst hatching

The successful hatching of the embryos is thought to be a key event in the implantation process. One reason for the low implantation rate that has been suggested is the limited ability of blastocysts to hatch from the zona pellucida. Suboptimal culture conditions might induce the hardening of zona pellucida, which could limit the hatching ability of embryos [176]. To help embryos hatching from their zona pellucida during blastocyst expansion, different types of assisted hatching have been developed, including mechanical partial zona dissection or zona drilling, chemical zona drilling with acidic Tyrode's solution, and the laser dissection technique [177]. However, there is controversy about the benefit of assisted hatching on the improvement of the implantation rate and pregnancy rate. Some agreed as to the benefit of assisted hatching in women with advanced age, in women with repeated IVF-ET failure [178].

The degree of zona pellucida thickness variation of the transferred embryos has been found to exhibit a strong correlation with clinical pregnancy outcome following IVF-ET treatment and to be important for embryo selection during clinical transfers [179,180]. Zona pellucida thickness variation and character were found to correlate with implantation. Implantation rates were found to range from 10% for embryos with uniform thickness to 29% with thin or irregular zona pellucida [181,182]. These reports suggested that patients transferred with embryos with thinner zonae had a better chance of successful implantation and pregnancy as compared to those transferred with embryos having thicker zonae. Other reports have proposed zona pellucida thickness variation as a reliable marker for selecting thawed as well as fresh human embryos for transfers [183,184]. Cohen [185] conducted a retrospective analysis of zona pellucida thickness of transferred embryos through video recordings and concluded that the variations in zona pellucida thickness rather than the zona pellucida thickness per se of the transferred embryos was a stronger predictor of the IVF-ET.

A significant linear relationship was reported to exist between the mean zona pellucida thickness of each patient and the maximum E_2 _level and an increasing one with the hMG dose. The authors found that the zona pellucida thickness was basically an individual feature that influenced the fertilization rate [186].

##### 3.1.5 Effect of supraphysiological estrogen levels on the ovaries [corpus luteum], pituitary, and hypothalamus

Abnormalities in the luteal phase have been shown in virtually all the stimulation protocols used in ovarian stimulation, on the hormonal, as well as on the endometrial level. All three aspects of a defective luteal phase, that is, a shortened luteal phase and/or low mid-luteal serum progesterone concentrations and/or abnormal endometrial histology, have been regularly observed in IVF-ET cycles. For that reason, luteal-phase supplementation with hCG or progesterone increases pregnancy rates, and its necessity has been well established, at least in GnRH-agonist cycles [187].

In the 'pre-agonists era', Edwards and Steptoe were the first to postulate luteal phase inadequacy resulting from ovarian stimulation as a cause of failure of IVF-ET cycles. With the introduction of the GnRH-agonist, used in ovarian stimulation cycles to avoid premature luteinizing hormone [LH] surge, luteal phase inadequacy was reported. A meta-analysis of different clinical trials demonstrated beneficial effects of luteal support when ovarian stimulation was carried out with human menopausal gonadotropins [hMG] in association with GnRHa [187].

Recently, GnRH antagonists have become available for clinical use. Whether GnRH antagonists induce down-regulation of pituitary GnRH receptors is still a subject of investigation [188]. It has been demonstrated that chronic administration of the GnRH antagonist Cetrorelix in rats causes an important down-regulation of pituitary GnRH receptors [189].

###### Direct effect on corpus luteum

Different estradiol receprors have been detected in human corpus luteum, indicating that estrogen might be a local regulator of corpus luteum function [190]. In the pre-agonist era, the alteration of the Estradiol/progesterone (E_2_/P) ratio was considered a main cause of luteal-phase inadequacy, possibly through the luteolytic action of E_2 _[5]

Estrogen is thought to exert a direct luteolytic action in primates as exogenous administration of E_2 _reduces progesterone concentrations during the luteal phase [191], probably via inhibiting the enzyme 3 beta-hydroxysteroid dehydrogenase, which is mandatory for progesterone synthesis [192]. Moreover, although the exact mechanism has not yet been established, estrogen may play a role in the regulation of proteins involved in the process of luteal-cell apoptosis [193].

Normal corpus luteum function is dependent on the proper function of the pituitary gland and hypothalamus. Adequate luteinizing hormone [LH] surge during ovulation and continuous tonic LH pulses during the luteal phase are necessary for the proper development of the corpus luteum. Corpus luteum dysfunction due to the effect of ovarian stimulation on the pituitary gland and hypothalamus will be discussed later.

###### Effect on the pituitary gland and hypothalamus

Normal corpus luteum function requires optimal follicular development in the follicular phase, especially follicle stimulating hormone (FSH) stimulation, adequate luteinizing hormone (LH) surge during ovulation and continuous tonic LH pulses during the luteal phase. In turn, the normal luteal phase is characterized by an optimal hormonal environment and adequate endometrial secretory transformation. As many factors contribute to a normal corpus luteum function, any alteration might exert a deleterious effect on the final target, the endometrium, leading to embryo/endometrial asynchrony [5]. Luteal phase insufficiency due to corpus luteum defects and LH suppression is known to be associated with failure to achieve and maintain pregnancy [194].

###### Effect on LH secretion

The lifespan and steroidogenic capacity of the human corpus luteum is dependent on continuous tonic luteinizing hormone secretion as well as healthy adequate gonadotropins surge. Feedback mechanisms from ovarian steroids and GnRH pulses regulate LH secretion during the luteal phase, but a number of autocrine and paracrine factors within the ovary might also play a role in controlling corpus luteum function [5].

####### Effect on LH surge

It is obvious that during COH for ART, the prevention of endogenous LH surge is mandatory to avoid the occurrence of premature LH surge as well as for timing of oocyte retrieval.

However, during ovarian stimulation cycles in which no pituitary suppression is used, there are data that suggest a defective endogenous gonadotropins surge. The gonadotropins surge is an event crucial for final oocyte maturation, ovulation, and subsequently for corpus luteum function. The duration of the LH peak seems to be more important than its amplitude for the induction of ovulation [195]. Ovulation induction by hCG is not physiological; the absence of an FSH surge, and the long duration of LH activity associated with hCG action, would contribute to some of the luteal phase abnormalities [196].

Messinis demonstrated that an attenuated LH surge is obtained in normally cycling women during superovulation induction with sequential clomiphene/hMG treatment. The peak values and the duration of the LH surge to have significant negative correlations with the plasma E_2 _levels, the number of follicles, and the total follicular fluid volume aspirated at laparoscopy. This suggests that during superovulation induction for IVF-ET, the endogenous LH surge is attenuated by factors, which are related to the degree of ovarian hyperstimulation [197].

As shown above, the induction of superovulation in women with human gonadotropins may result in blockage of the endogenous LH surge, but the reasons for this are not known. A high number of small follicles have been suggested to have a suppressive effect on both tonic and mid-cycle gonadotropins secretion [198].

####### Effect of abnormal LH surge on nuclear maturation of the egg

A timely LH surge of adequate amplitude and sufficient duration is important to bring about rapid and complex cellular differentiation, resulting in cascades of tightly coupled biochemical events, which initiate oocyte maturation, ovulation, and corpus luteum formation [199]. It is known that a midcycle LH surge of sufficiently high amplitude and duration is important for both nuclear and cytoplasmic maturation, which ensure the normal fertilization and developing potential of oocytes [199]. For PMSG-hyperstimulated rats, higher doses of hCG are required to completely ovulate the expanded cohort of preovulatory follicles [200].

####### Effect on LH tonic pulse during the luteal phase

Low luteal LH levels have been described after human menopausal gonadotropins treatment^6 ^and after GnRH-agonist treatment or after GnRH-antagonist treatment. These low, almost undetectable, luteal LH levels may not be able to support corpus luteum. As a result, a shortened luteal phase and low mid-luteal progesterone concentrations have been described in cycles stimulated with the association either of a GnRH agonist or a GnRH antagonist [195,196].

Supraphysiological progesterone serum concentrations may also interfere with the pituitary's luteinizing hormone secretion by disturbing the feedback control mechanisms and may result in a reduction of the LH serum levels [195,196]. Progesterone modulates LH secretion during the luteal phase by influencing the LH pulse amplitude and pituitary release of LH [201]. A longer exposure to progesterone or the combined action of estrogen and progesterone decreases LH release [302]. As ovarian stimulation results in supraphysiological steroid serum concentrations, these high steroid levels may adversely affect LH secretion via a long-loop feedback mechanism. In turn, disturbed LH secretion may induce a luteal-phase defect with premature luteolysis, low progesterone levels and shortened luteal phase. It might therefore be hypothesized that deviation from the normal hormonal environment could be a prevalent effect of ovarian stimulation in the luteal phase; despite the use of different stimulation protocols [7]. This might be a possible explanation of the observation that in natural cycle, luteal-phase length was normal after GnRH-antagonist treatment [203]. However, in GnRH-antagonist cycles and after minimal ovarian stimulation, luteal-phase length was normal despite an abnormal endocrine profile [204].

##### 3.1.6 Other probable effects of supraphysiological estrogen levels

There are other less defined probable mechanisms through which supraphysiological estrogen levels may cause adverse effects on the outcome of infertility treatment.

###### (A) Leptin-mediated effect

Recently, an important role for the leptin, the secretory product of adipocytes, in reproductive medicine has emerged and it is interesting to discuss in brief a possible link between leptin, induction of ovulation and aromatase inhibitors.

Soon after its discovery in the early 1990s, it was recognized that leptin played a significant role in reproduction, providing a critical link between metabolic state and fertility. It now appears that leptin may have an important role in both normal ovarian physiology and pathophysiology. Research has revealed that a minimum level of leptin stimulation is required for maintenance of fertility in animals and humans. Conversely, elevated leptin levels may impair fertility [205].

Leptin is a secretory product of adipocytes that correlates significantly with the body mass index with increased levels in obese women [206]. It can influence reproduction through central (on GnRH neural system) and peripheral (on the ovary directly) mechanisms [205]. A positive correlation has been found between leptin and BMI, as well as between leptin and testosterone in women with polycystic ovarian syndrome (PCOS) [207].

Numerous studies have shown that circulating leptin concentrations rise in parallel with E_2 _during ovarian stimulation [208-213]. These changes are not likely to be a direct action of FSH because FSH decreases as E_2 _and leptin rise during natural cycles, and high FSH levels experienced during ovarian stimulation simulate endogenous levels in postmenopausal women, who have lower serum leptin concentrations than premenopausal women [205]. Bützow [206] found that the larger the increase in serum leptin concentrations during FSH stimulation, the poorer the ovarian response in terms of number of follicles and retrieved oocytes. Moreover, higher serum leptin levels were found in oligo- and amenorrheic women who failed to respond to clomiphene therapy [214].

The effect of body weight on outcomes of assisted reproduction has been investigated. Fedorcsak [215] reported that among patients who conceived, overweight patients (BMI >25) had fewer oocytes retrieved, a higher miscarriage rate, and lower live birth rate. In a much larger retrospective study that included 8822 embryo transfer cycles, the cumulative pregnancy rate progressively decreased as BMI increased from <25 to >35 [216].

Higher follicular fluid leptin concentrations correlated with lower intrafollicular oxygen concentration [pO_2_], [217] a condition that negatively impacts oocyte developmental competence [218] with a direct evidence of a relationship between leptin levels and ART outcome reported by Mantzoros [219] who found significantly lower follicular fluid leptin concentrations in women who became pregnant within three cycles of IVF-ET or gamete intrafallopian transfer (GIFT). More recently, a significant negative correlation between non-fasting serum leptin levels measured at the beginning of FSH stimulation and pregnancy success in women undergoing first attempt IVF-ET cycles was reported [220]. Moreover, as leptin receptors are expressed in the human endometrium, [221] a role for leptin in endometrial receptivity cannot be excluded. These results imply that elevated leptin may be a key factor in obesity-related fertility problems, and conversely that elevated leptin may negatively impact fertility independently of body mass. Fewer good embryos and lower implantation rate suggest that elevated leptin impairs oocyte developmental competence and/or early cleavage stage embryo development, possibly via direct actions on the follicle [205].

E_2 _has been reported to increase leptin mRNA expression. In human adipose tissue culture, E_2 _stimulated leptin secretion in women but not in men [222]. In women, E_2 _was found to increase ob mRNA expression and leptin release. Moreover, in adipose tissue of women, the estrogen precursors; testosterone and dehydroepiandrosterone also induced an increase in leptin secretion, an effect that was prevented by the aromatase inhibitor letrozole. Moreover, the stimulatory effect of E_2 _observed in women was antagonized by the antiestrogen ICI182780 [223].

###### (B) Coagulation system

Estrogen has been pointed out as a pre-thrombotic factor. It has long been established that both pregnancy and oral contraceptive use have resulted in an increased level of many coagulation parameters. Generally, this has been thought to be a result of the estrogen component. Although it has been well established that long-term exposure to exogenous contraceptive steroids can have a promoting influence on the potential for thrombosis in women, it is less clear what role high levels of endogenous steroids might play. Undefined coagulation abnormalities were reported after hMG- and hCG-induced hyperstimulation of the ovary in several women [224-227]. Kim et al., [225] noted a large increase in fibrinogen after hMG treatment, accompanied by "significant increases" in the prothrombin time. However, the statistically significant activation of clotting factors occurring during controlled ovarian hyperstimulation is usually not accompanied by clinically significant coagulation disorders that may be explained by either of the following: (1) the increased levels of clotting activity were still "within normal limits"; (2) none of the patients had any conditions known to predispose to coagulopathies (history of coagulopathies, phlebitis, damaged or compromised endothelial cells, etc.); or (3) a combination of the two [226]. However, such subtle coagulation disturbances may exert an adverse effect on endometrial receptivity and the early development of the placenta by affecting the microcirculation.

Two case reports were published of activated protein C-resistant women who suffered a thrombotic event during IVF-ET treatment [227,228]. Curvers [229] reported that the only coagulation parameter that changed considerably during IVF-ET treatment was the activated protein C (APC). In their study, the authors observed that hyperstimulation, i.e. high estrogen levels, induce APC resistance, and that under these conditions both the absolute values and the changes in the APC and the estrogen levels (hyperstimulation-baseline) correlate significantly. Prior to that study, it was reported that high estrogen levels were not associated with APC resistance [230,231]. However, the APC resistance test used in these studies, which is based on quantification of the effect of APC on the clotting of plasma initiated via the intrinsic coagulation pathway, is not very sensitive to changes in sex hormones [232]. On the other hand, Curvers et al. [232], used an assay that quantifies down-regulation of extrinsic coagulation by APC that is particularly sensitive to hormonal changes in women [232].

We think it is possible that the reported effect of ovarian stimulation-associated supraphysiological levels of estrogen on the coagulation system, though being modest, may contribute at least partially to the reduced implantation rate observed after assisted reproduction. This could be due to an impact on the microcirculation in the endometrium that could affect the early stages of the implantation as well as the early development of the placenta. However, more studies are needed to support this hypothesis.

###### (C) Tubes and tubal transfer

####### Effect on the oviductal environment

Oviducts are biologically active, providing an environment that sustains and enhances fertilization during early embryonic development as the embryo travels toward the uterine cavity. Following superovulation, the fluid from the oviduct seems to impair embryo development. Furthermore, a stimulated oviductal environment has also been shown to have a negative influence on the implantation capacity of mouse embryos [233].

####### Superovulation in the mouse was described as a model for intra-uterine growth retardation

Superovulation is associated with a slower preimplantation embryo development, a later and impaired implantation and a prolonged gestation [234]. This suggests that the oviductal milieu rather than the embryo quality are responsible for the adverse effects observed after superovulation. The stimulated oviductal environment impairs the developmental capacity of embryos in comparison with untreated pseudopregnant females. In-vitro culture is also suboptimal but better than the stimulated oviductal environment. However, a detrimental effect of hormonal stimulation upon the oviductal environment has not yet been demonstrated in the human. A possible potential negative effect, however, is not contradicted by observations reporting a higher pregnancy rate after ovarian stimulation and IUI compared to insemination alone [235]. In the human, there are observations that GIFT results in higher pregnancy rates in comparison with IVF-ET [236,237]. It is difficult to interpret these data since the embryos were exposed longer to the possible deleterious stimulated oviductal environment-using GIFT whereas in IVF-ET, possible "suboptimal" culture conditions as opposed to in-vivo conditions were used. Since the introduction of sequential culture media, incorporating amino acids, vitamins and growth factors, the in-vitro culture of embryos has been improved, resulting in implantation rates of >50% per transferred blastocyst [238]. Confirmation of these data in a trial would result in higher pregnancy rates than ever reported for GIFT [239].

####### Effect on the oviductal embryo transfer

Akira [240] has found that ovarian stimulation was associated with accelerated oviductal embryo transport. The authors have concluded that increased implantation failure in superovulated rats may result from the accelerated embryo transport resulting from elevated E_2_/P ratio. Accelerated oocyte/embryo transfer has been postulated to be the mechanism behind which high doses of estrogen work as postcoital emergency contraception as explained later.

####### Post-coital contraception with estrogen

The achievement of very high levels of estrogen by administering exogenous estrogen has been suggested for postcoital contraception as early as the 1960s, when high-dose estrogen was identified as a highly effective emergency contraceptive [241].

Greenwald [242] compared the response of the rabbit, rat, mouse, hamster, and guinea pig, to a single post-coital injection of E_2 _cyclopentylpropionate and showed that post-coital treatment with estrogens caused either tube locking of embryos or accelerated transport to the uterus. Although other effects were also detected, the alteration in oviductal transport accounted for the contraceptive effect. Embryos that entered the uterus prematurely were expelled whereas whose sojourn through the oviduct was prolonged, degenerated.

Other investigators found that a single injection of E_2 _was given at different times after coitus revealed that a wide range of effectiveness can be achieved and suggested different mechanisms can account for the contraceptive effect when the same steroid is given at different times post-coitus [243,244]. Some believed the main target was the endometrium where they observed stromal edema, hemorrhage, and loss of decidua, all of which was considered unsuitable for implantation. Administering high dose of estrogen in the preovulatory phase was found to depress endometrial growth and angiogenesis through a negative influence on the vascular endothelial growth factor [245]. This suggested that estrogens might interfere with endometrial receptivity even if given before ovulation.

Other mechanisms for the contraceptive effect of post-coital estrogen were found to operate in monkeys. If given in the follicular phase, so as to advance the preovulatory increment in plasma estrogen, they evoke a premature LH surge that does not trigger ovulation and the formation of a functional corpus luteum, and the spontaneous LH surge is delayed or suppressed [246]. ER present in the granulosa cells of antral and preovulatory follicles and in luteal cells [247] allow for a diversity of effects in the ovary. In rhesus monkey, supraphysiological doses of estrogen given in the mid or late follicular phase induce atresia or luteinization without rupture of the dominant follicle, reduce the viability of granulosa cells, reduce the synthesis of E_2 _and progesterone and are detrimental to the oocyte [248].

Whatever the mechanism of action of high dose of estrogen for emergency contraception is, success in preventing pregnancy provides another evidence of the toxic effect of the supraphysiological estrogen levels fertilization, implantation and early development of the embryo which result in failure of achieving pregnancy.

###### [D] Effect on other factors involved in decidualization and early developing placenta

####### Paracrine factors

The importance of paracrine factors in mediating the cellular and biochemical changes involved in embryo implantation has been recognized. Many growth factors and cytokines, such as inhibins and activins, whose expression is generally limited to developmental and pathological states, are produced by actively remodeling endometrial cells, and play crucial roles in regulating endometrial cell function. Example of these factors includes the inhibin and activin family in the paracrine regulation of endometrial receptivity, decidualization and implantation. Estrogen is known to play an important role in regulation of these factors as discussed by Jones [249].

####### Role of Calcitonin

The peptide hormone calcitonin is currently being evaluated as a potential marker of the fertile human endometrium. In ovariectomized animals, it has been shown that administration of estrogen together with progesterone inhibits progesterone-mediated calcitonin gene induction.^29 ^Such antagonistic interactions between estrogen and progesterone pathways have been documented previously in breast and uterine cells. It has been proposed that these phenomena reflect transcriptional cross talk occurring between estrogen and PR co-expressed in the same target tissue. A complex interplay of the two ovarian hormones, progesterone and estrogen, in the uterine milieu is believed to be critical for optimal calcitonin gene expression [250,251].

#### 3.2 Effect of the medications used for ovarian stimulation

##### 3.2.1 Clomiphene citrate

In spite of the high ovulation rate with the use of clomiphene citrate (around 50–90%), the pregnancy rate is much lower (around 20–40%) [252-254]. Moreover, there is a higher than expected incidence of miscarriage in conception cycles following clomiphene citrate treatment [255]. Such discrepancy is believed to be due to the peripheral antiestrogenic effect of clomiphene citrate, particularly at the level of the cervical mucus [256,257] and endometrium, [258,259]. The persistence of the zu-isomer of clomiphene citrate in the body due to its long half-life (several weeks) and slow clearance adds to the accumulation of the antiestrogenic effects over subsequent cycles of administration [260,261]. There is also evidence of a direct harmful effect of a high concentration of clomiphene citrate and its isomers on fertilization, and on early mouse [262] and rabbit [263] embryo development. Such effects, however, were not confirmed in other studies [264,265]. There is still some controversy concerning a direct effect on the quality of oocytes associated with clomiphene citrate treatment [266,267]. Decreased uterine blood flow during the early luteal phase and the peri-implantation stage is another explanation for the poor outcome of clomiphene citrate treatment [268]. Other investigators have suggested the presence of other unrecognized infertility factors [269,270].

##### 3.2.2 Gonadotropins

The existence of nongonadal gonadotropins (FSH, LH/hCG) receptors was first suggested by Ziecik [270] who conducted binding studies in the porcine uterus. Subsequently, nongonadal receptors were found in human tissues, including endometrium, myometrium, fallopian tube, umbilical cord, and brain, by using a variety of techniques, including immunohistochemistry; Northern, Western, and ligand blotting; and in situ hybridization [271-274]. This would suggest a possible direct action of the gonadotropins on the uterus and involvement in the endometrial development, implantation and establishment of pregnancy [275].

High concentrations of exogenous gonadotropins used for hyperstimulation of folliculogenesis were shown to be detrimental to oocyte and embryo development in many animal species. This is believed to be due to the associated supraphysiological E_2 _levels and other possible undetermined factors associated with ovarian stimulation. However, a direct effect of gonadotropins cannot be ruled out. It has been reported that early embryo loss due to superovulation could be rescued by an injection of goat antiserum against PMSG [276].

###### Effect of gonadotropins on LH surge

It has been suggested that during ovarian stimulation, the supraphysiological FSH levels that persist into the late follicular phase, thereby overriding selection of the single dominant follicle of the natural cycle, secretion of an ovarian factor(s) blocks estrogen-induced LH surges [277]. Accumulated evidence has indicated that the ovaries produce another non-steroidal substance, named "gonadotropins surge-attenuating factor" (GnSAF), which may play a role in the control of the midcycle luteinizing hormone surge in women [278-281]. Although GnSAF activity is present during superovulation induction, it is still unclear whether this factor plays a physiological role during the normal menstrual cycle. Treatment with FSH initially attenuated the response of LH to GnRH via the production of GnSAF from the ovaries, while around the midfollicular phase, the rising concentrations of E_2 _were able to overcome the attenuating effect of GnSAF and increase pituitary sensitivity to GnRH. The increased pituitary sensitivity in the midfollicular phase of the FSH-treated cycles, however, was not further enhanced in the late follicular phase despite the continuous rise in E_2 _values [282]. It is suggested that eventually GnSAF was able to overcome the sensitizing effect of E_2_

###### Effect of gonadotropins on chromosomal development

A dose-response relationship between the PMSG dose and the incidence of polyploidy in the CD-1 mouse has been reported with the level of polyploidy rising from 2.9% with 10 IU PMSG to 10.5% with 15 IU PMSG, in the zygot stage. Both a disturbance at maturation division and an error at fertilization were the cause of polyploidy [22]. Whether this is the direct effect of PMSG or the resultant ovarian stimulation is not known.

##### 3.2.3 GnRH analogues

GnRH plays a pivotal role in the control of female reproduction and is secreted by hypothalamic neurons in a pulsatile way. It binds to specific receptors on pituitary gonadotrophs, which is followed by the secretion of the gonadotropins, LH and FSH, which regulate steroidogenesis and gametogenesis in the ovary [283].

GnRH analogues are able to suppress gonadotropins release and, subsequently, gonadal function. This is the basis for their clinical application in ovarian stimulation. Several agonistic or antagonistic GnRH analogues have been developed for this purpose [284].

###### Effect of GnRH analogues on ovarian steroidogenesis and corpus luteum function

GnRH receptors and GnRH receptor mRNA were found to be expressed in human granulosa-luteal cells GnRH receptors have been found in the luteinized human granulosa cells, and a possible direct effect of GnRH on ovarian steroidogenesis has been suggested [485,486].

Although there are many studies that investigated the possible direct effect of various GnRH agonists on ovarian steroidogenesis and hence the corpus luteum, there is no consensus of the effects in the human ovary. Also, there is little information concerning the direct effect of GnRH antagonists on ovarian steroidogenesis [487]. A few studies that looked at the effect of GnRH agonist treatments in vivo on in vitro steroidogenesis by human luteinized granulosa cells, found an impairment of progesterone production [287-290]. Minaretzis [291] have reported on the effect of the GnRH antagonist Nal-Glu compared with GnRH agonist leuprolide acetate treatment of patients undergoing controlled ovarian hyperstimulation. The progesterone production in human luteinized granulosa cells cultures was not different between the two groups. Both GnRH antagonists had no effect on basal or hCG-induced E_2 _or progesterone production by granulose lutein cells, independent of whether the cells were exposed to the compounds in vitro or in vivo. However, an in-vitro study demonstrated an inhibitory effect of GnRH antagonist on gonadal steroid secretion [292]. Pellicer and Miro who found that GnRH agonist exposure in vivo may affect human luteinized granulosa cells function in vitro. They compared the progesterone accumulation in human luteinized granulosa cells cultures between patients treated with clomiphene citrate/gonadotropins and GnRH agonist/gonadotropins. Granulosa cells obtained from patients treated with the GnRH agonist had lower progesterone production than cells isolated from women treated with clomiphene citrate/gonadotropins [293]. However, Minaretzis compared the human luteinized granulosa cells steroidogenesis in vitro from Nal-Glu and leuprolide acetate-treated women respectively. They reported that basal and gonadotropins-stimulated progesterone secretion was similar in the two treatment groups [291].

Weiss et al., found that in vivo treatment with triptorelin or the two GnRH antagonists, cetrorelix and ganirelix; did not have an effect on spontaneous or hCG-stimulated steroidogenesis. The authors also performed in vitro treatments with triptorelin, cetrorelix and ganirelix for up to 96 hours and did not find any effect of these treatments on basal or hCG-stimulated steroid production [287].

In all prior reports, which studied the effect of GnRH agonist treatment on ovarian steroidogenesis, only the effect of hCG on progesterone secretion by human luteinized granulose cells was investigated. Recently, we examined the effect of two known physiologic stimuli of ovarian steroidogenesis, hCG and insulin. We found that GnRH agonist treatment affected both the basal and hCG-stimulated progesterone production by human luteinized granulosa cells but the pattern of insulin-stimulated progesterone secretion was not affected. Because insulin and hCG may share common pathways beyond but not at the level of receptor activation, we hypothesize that in-vivo GnRH agonist might affect the expression and/or activation of LH receptors. In our study, we found that human luteinized granulosa cells from patients treated with GnRH antagonist responded to hCG in a fashion generally consistent with previously reported hCG-stimulated human luteinized granulosa cells progesterone production [294]. In addition, the basal progesterone production and absolute levels of insulin-stimulated progesterone production were both significantly higher than observed following GnRH agonist treatment. In contrast, human luteinized granulosa cells previously exposed in vivo to GnRH agonist had a blunted P response to hCG. Our results, therefore, support the hypothesis that GnRH agonists may have a direct negative effect on human ovarian steroidogenesis by the corpus luteum and suggest that luteal function may be less affected during IVF-ET cycles when GnRH antagonist is used. However, in another study, GnRH antagonist therapy in women undergoing ovarian stimulation was found to be associated with a significant effect on ovarian follicular steroidogenesis. The authors found the mean follicular fluid E_2 _concentration significantly lower in patients treated with GnRH antagonist than in those treated with GnRH agonist. However, no significant differences were found between groups in follicular fluid progesterone concentrations [295]. However, further studies are required to investigate the effect of GnRH agonist and antagonist on gonadotropins receptors and different enzymes involved in ovarian steroidogenesis by the corpus luteum.

###### Effect on LH secretion

Because pituitary LH secretion is dependent on GnRH stimulation and feedback mechanisms from ovarian steroids, any alteration may be deleterious. In cycles using GnRH agonists for ovarian stimulation, a significant drop in mid-luteal progesterone concentrations was observed, consistent with corpus luteum insufficiency. Long-term GnRH-agonist administration has been associated with a profound desensitization of the pituitary cells. In fact, studies on pituitary gonadotropins secretory capacity after GnRH-agonist treatment have indicated that this remains impaired for at least 14 days after the discontinuation of the GnRH-agonist and for the whole length of the luteal phase [296]. In a randomized trial, it was also demonstrated that, despite the early cessation of the GnRH agonist in the follicular phase, luteal-phase characteristics were abnormal [297].

GnRH-antagonist treatment has been shown to be effective in blocking the LH surge. GnRH antagonists bind competitively to pituitary GnRH receptors and cause an immediate inhibition of gonadotropins release. In contrast to GnRH agonists, it was suggested that treatment with GnRH antagonists might not adversely affect luteal LH secretion, since the pituitary maintains its responsiveness to the endogenous GnRH stimulus [298]. A normal luteal phase, in terms of duration and serum progesterone concentrations, was observed in natural cycles in which an antagonist was administered to prevent the LH surge [299]. However, data on the luteal phase from unsupplemented cycles after antagonist administration are limited as a result of the small number of patients involved and are rather controversial. Four out of six patients had either a shortened luteal phase or low progesterone concentrations in cycles stimulated with HMG and the GnRH antagonist Cetrorelix and receiving no luteal-phase supplementation. However, with or without luteal-phase supplementation, luteal LH levels were low, indicating that another mechanism might be involved [299,300].

##### 3.2.4 Human chorionic gonadotropins

Supraphysiological steroid serum concentrations may interfere with LH secretion via long-loop feedback, but, additionally, the exogenously administered hCG might amplify LH secretion arrest via a second short-loop negative feedback. Although in the monkeys such a negative feedback exists [301] there is a debate in the literature about its existence in humans, with some of the studies supporting this hypothesis [302] and others not [303,304]. Nevertheless, findings from in-vitro studies further support this idea. GT1-7 neurons, which are morphologically and functionally similar to GnRH neurons, were found to contain LH/hCG receptors. In addition, exogenously administered hCG was found to decrease the expression of GnRH receptor gene in GT1-7 cells or GnRH secretion in immortalized GnRH neurons [305].

### 4 Measure to improve treatment outcome after ovarian stimulation and assisted reproduction

Even if the results of reproductive medicine have improved in terms of numbers of pregnancies, it is still striking that it is necessary to use stimulation which sometimes leads to hyperstimulation and multiple pregnancies, that embryo development in vitro is still limited, that implantation only occurs for 15–20% of transferred embryos and this ratio has not changed significantly along the last 25 years. We still need to improve techniques to gain pregnancy rates approaching 50% per embryo. During assisted reproduction technology interventions, controlled ovarian stimulation is used to obtain several oocytes in attempts to increase the likelihood of having at least one developmentally competent embryo available for transfer. However, current techniques for identifying the competent embryo[s] are by no means perfect. These limitations, coupled with pressures to maximize the chance of pregnancy, typically result in the transfer of multiple embryos. Not surprisingly, this practice has resulted in an unacceptably high rate of multiple pregnancies arising from IVF-ET. During the last few years, concerted efforts have focused on reducing these rates by restricting the number of embryos to transfer [306].

In order to fulfill this task, several approaches have been suggested to improve the outcome of treatment after ovarian stimulation and assisted reproduction. Table 3 summarizes the different approaches including the two main approaches tried so far i.e. optimizing ovarian stimulation protocols and improving embryology techniques. A third approach we suggest i.e. the use of aromatase inhibitors constitutes the third and most recent approach.

**Table 3:** Various approaches to improve treatment outcome after ovarian stimulation:

(1) Approaches involving ovarian stimulation protocols:
A: Reduce intensity of ovarian stimulation
- No stimulation: natural cycle
- Minimal stimulation
- Step-down protocol
B: The application of new medications
- Recombinant FSH
- Recombinant LH
- GnRH antagonists
C: The use of adjuvant medication
- Insulin sensitizers
- Corticosteroids
- Other medications
(2) Improving embryology techniques
- Improving embryo culture conditions, selection and extending embryo culture (blastocyst transfer)
- Preimplantation genetic diagnosis
- In vitro maturation
- Micromanipulation including: ICSI, assisted hatching, mitochondrial injection and cytoplasmic and nuclear transfer.

(3) The use of aromatase inhibitors for
- Improving implantation rates
- Reducing FSH dose required for ovarian stimulation:
- Other possible benefits:
* Improving response to FSH stimulation in poor responders
* Preventing premature endogenous gonadotropin surge
* Reducing the risk of severe ovarian hyperstimulation

We suggest a THIRD novel approach that we believe it may carry a hope for improving the outcome of treatment after ovarian stimulation and assisted reproduction. This approach involves the use of aromatase inhibitors.

#### 4.1 Modified ovarian stimulation protocols

##### 4.1.1 Reducing the intensity of ovarian stimulation protocols

Different approaches have been suggested to improve the treatment outcome during assisted reproduction by reducing the intensity of ovarian stimulation that would reduce the high estrogen levels [307]. These approaches include no-stimulation (natural cycle IVF-ET), minimal stimulation IVF-ET cycles, step-down protocols, FSH coasting and in vitro maturation.

However, all these measures are associated with the major drawback of affecting the main goal of ovarian stimulation, which is the achievement of a suitable number of embryos when a good number of oocytes retrieved.

###### Natural cycle IVF-ET

The first IVF-ET treatment in the literature was a natural cycle IVF-ET. Since then natural cycle IVF-ET has been largely replaced by IVF-ET with ovarian stimulation. Natural cycle IVF-ET has several advantages including a close to zero multiple pregnancy rate, and a zero risk of ovarian hyperstimulation syndrome as well as being less time consuming, physically and emotionally less demanding for patients, and cheaper than stimulated IVF-ET. However, it seems to be less effective. Unfortunately, good quality randomized controlled trials and formal cost-effectiveness analyses are lacking. Pelinck reviewed 20 selected studies that comprised a total of 1800 cycles of natural cycle IVF-ET, resulting in 819 embryo transfers (45.5% per cycle) and 129 ongoing pregnancies (7.2% per cycle and 15.8% per embryo transfer). The authors concluded that: in spite of being a low-risk, low-cost and patient-friendly procedure, high cancellation rates because of premature LH rise and premature ovulations hamper efficacy of natural cycle IVF-ET and that a randomized controlled trial comparing natural cycle IVF-ET with current standard treatment strategies is warranted. [27].

The cumulative live birth rates after four cycles of treatment was reported to be 32% which is comparable with the value of 34% for women having conventional IVF-ET treatment [308]. However, there is enough data on cumulative live birth rates following conventional IVF-ET, which is likely that current cumulative rates for IVF-ET will be higher than previously reported.

In the implantation rate per embryo after natural cycle IVF-ET was found quite acceptable up to 50%. Also, the fertilization rate was found to be 100% for the oocytes retrieved from the mature follicles (as reviewed by [27] Pelinck). We believe that the significantly better oocyte quality (100% fertilization rate) as well as better implantation rate (about 50%), reflect clearly the value of avoiding the deleterious effects of ovarian stimulation on the treatment outcome. Moreover, there is an important point to mention regarding the success rates of natural IVF-ET cycles, which is the bias in selecting patients who underwent natural IVF-ET cycles. Many studies included patients with unfavorable outcome such as poor responders, old age and repeated IVF-ET failures.

###### Minimal stimulation IVF-ET

Minimal stimulation for IVF-ET that is less expensive than full stimulation and minimizes monitoring and patient discomfort has been described for almost 10 years and found to be associated with acceptable pregnancy rates and was suggested as an attractive alternative to select patients undergoing IVF-ET [309]. This protocol involves the use of CC plus FSH alone or in conjunction with GnRH antagonist to prevent premature LH surge [310]. It is expected that the minimal-stimulation regimens produce fewer oocytes, fewer embryos available after fertilization, and fewer excess embryos available for cryopreservation. This leads to the main drawback of the minimal-stimulation regimen, which is the lower total reproductive potential (pregnancies resulting from fresh and frozen-thawed embryos resulting from a single stimulation cycle) [311].

###### FSH step-down and coasting protocols

Other measures have been tried by decreasing the FSH dose (step down protocol) to improve endometrial receptivity. With the use of a step-down regimen with FSH in high responders, uterine receptivity was believed to improve secondary to lowering E_2 _levels during the preimplantation period [312].

Coasting or withholding FSH injection for a period of time has been suggested in patients at substantial risk for the development of severe hyperstimulation syndrome. Coasting in a studied subset of IVF-ET patients did not adversely affect cycle outcome parameters when it was not prolonged [313].

##### 4.1.2 Application of new medications for ovarian stimulation

The ongoing development of more effective and safer pharmacological compounds is driving fundamental changes in medicine [314]. Along the last decade several new medications have been developed for application in ovarian stimulation and assisted reproduction including medications prepared by the new recombinant technology [recombinant FSH and LH] and GnRH antagonists as well as other medications.

###### Recombinant FSH

The introduction of recombinant FSH into the clinical management of patients suffering from infertility appears to be associated with several treatment benefits when compared with urinary human menopausal gonadotropins

The fact that rFSH preparations have batch-to-batch consistency, are free from urinary protein contaminants and have the potential to be produced in limitless quantities is advantageous. The question whether newer, more pure FSH products are beneficial from the clinical perspective, has not been settled beyond reasonable doubt. The price of rFSH has been reported to be three times as high as the price of the former FSH preparations [315].

Although both FSH and LH are required for normal follicular growth and maturation, the precise role of LH is at present still uncertain. Since it was shown in the late 1980s that too high a concentration of LH might have a negative effect on fertilization and embryo quality, the idea arose that pure FSH preparations might be superior to hMG preparations for patients with endogenous LH production [316]. This hypothesis was tested in several clinical trials comparing uFSH with hMG with respect to pregnancy rates per IVF-ET treatment cycle. Statistical significance was not reached in any of these individual studies [316,317]. However, in 1995 a meta-analysis by Daya was published, which included eight studies and demonstrated a significant difference in favor of uFSH [316]. A few years later this finding was contradicted by a meta-analysis by Agrawal who argued that meta-analyses should take into account the different pituitary desensitization protocols used [318]. When pooling together 11 trials with the most commonly used GnRH agonist protocol [the long protocol], the overall odds ratio for comparing FSH and HMG was not significant. Although this meta-analysis was criticized on the issue of study selection bias, re-analysis following the inclusion and exclusion of selected studies did not change the overall results of the study [316,318,319].

The question whether rFSH also leads to more clinical pregnancies per IVF-ET cycle has been addressed by several clinical trials, none of which reached statistical significance. However, two meta-analyses pooling together the results of several trials did show significant treatment effects in favor of rFSH [320,321]. The interpretation of these meta-analyses is still debated, since they compare two types of rFSH (the alpha and beta variant), as well as different types of urinary FSH (uFSH and uFSH-HP, and in Out's meta-analysis one study with HMG was also included). In Daya's analysis, which received Cochrane status, an absolute increase in pregnancy rates of 3.7% (95% confidence interval [CI] 0.5–6.9%) was demonstrated comparing rFSH with uFSH/uFSH-HP.

Data on the third possible comparison, between rFSH and hMG, are scarce. The studies that have been conducted so far found no statistically significant differences with respect to ongoing pregnancy rates [322-324]. The evidence available at this time, comparing the different FSH containing gonadotropins preparations, concerns their usage in IVF-ET. However, almost half of all gonadotropins are used in other settings, such as IUI. Very little data are available comparing the preparations for this indication. With respect to adverse effects of exogenous FSH administration, no significant differences have been found between products. The main risk associated with the use of FSH containing gonadotropins products is the development of the ovarian hyperstimulation syndrome (OHSS). The incidence of OHSS does not differ between products [321-324].

###### Recombinant LH

hCG has been used as a surrogate LH surge because of the degree of homology between the two hormones. The major differences between the two hormones include the sequence of the β-subunit, the regulation of the secretion of the two hormones, and the pharmacokinetics of clearance of hCG as opposed to LH [325,326].

hCG has a slower plasma metabolic clearance, which consists of a rapid phase in the first 5–9 h following IM administration and a slower phase in the 1–1.3 days after administration. After 36 h, the calculated half-life of hCG was 2.32 days, as compared with LH, for which estimates have ranged from 1 h [326] to 3–5 h [327,328]. By day 10 after administration, less than 10% of the originally administered hCG was measurable [329]. However, the long serum half-life of hCG is likely to be an undesirable characteristic in clinical practice.

Until recently, there was no other biologic preparation that was as effective as hCG in triggering the final stage of ovulation. Recent developments using genetic engineering technologies and posttranscriptional biosynthesis have led to the production of rhLH, which has been available for use in clinical trials for several years [330]. The rhLH produced in vitro is purified and further formulated to yield a pharmaceutical preparation with very high specific immunoactivity and bioactivity. A crossover pharmacokinetic study has been performed in nonhuman primates to assess and compare pituitary, urinary, and rhLH [331]. After intravenous administration of pituitary and urinary-derived LH, and rhLH, mean concentration-time curves were parallel. The mean areas under the curve (AUC) for concentration by time after dose curves were similar, after correction for immunologic differences in dose. The mean clearance estimates and volume of distribution at steady state, distribution, and terminal half-lives were similar for all three types of LH. Furthermore, the results of studies in human volunteers show that rhLH and urinary-derived hLH have similar pharmacokinetic characteristics; both preparations presented an iv distribution half-life of 1.2 h and a terminal half-life of 10 h [332,333]. rhLH total body clearance was 2 L/h, with less than 5% of the dose being excreted by the kidneys. The steady state volume of distribution was approximately 8 L [332]. Furthermore, it has been demonstrated that co-administration of rhLH and rhFSH (follitropin α) does not modify their respective pharmacokinetic characteristics [332]. Finally, low doses of rhLH have been shown to promote ovarian steroidogenesis when follicular growth is induced with rhFSH in hypogonadotropic hypogonadal women [334].

The results show that a single dose of rhLH is effective in inducing final follicular maturation and early luteinization in IVF-ET and embryo transfer patients and is comparable with 5,000 IU u-hCG. A single dose of rhLH results in a highly significant reduction in OHSS compared with hCG. The dose of rhLH giving the highest efficacy to safety ratio was between 15,000 and 30,000 IU. This study has shown that the incidence of OHSS is significantly smaller when using a single dose of rhLH for induction of final maturation of follicles and oocytes in women treated for IVF-ET, compared with the use of 5,000 IU u-hCG or two doses of rhLH [335].

Chandrasekher [336] compared rhLH with u-hCG and pituitary hLH as an ovulatory stimulus in rhesus monkeys before IVF-ET. These investigators found that a single injection of 2,500 IU rhLH was as effective as 1,000 IU u-hCG in inducing oocyte maturation, oocyte fertilizability, and luteinization of granulosa cells. The data suggested that using a conversion factor of 2.5, a dose of 12,500 IU rhLH would be as effective as 5,000 IU u-hCG in humans. Another study (unpublished data) looked at the spontaneous LH surges in seven healthy female volunteers with normal menstrual cycles after a single injection of either 250 μg GnRH-agonist (buserelin) or 5,000 IU u-hCG administered when the dominant follicle reached a diameter of 17 mm. It was found that the mean C_max _of the spontaneous LH surge was 47 IU/L (95% CI, 28–65 IU/L) and the mean AUC was 1,019 IU h/L (95% CI, 718–1,320 IU h/L). Using rhLH pharmacokinetic characteristics defined in Phase I studies [332,333], it was calculated that, to obtain a C_max _and an AUC larger than the corresponding mean values observed in spontaneous surges in 95% of cases, single sc injections of between 14,000 IU and 25,000 IU rhLH should be used.

###### GnRH antagonists

GnRH antagonists do not induce an initial stimulation of gonadotropins release, but cause an immediate and rapid, reversible suppression of gonadotropins secretion. The principal mechanism of action of GnRH antagonists is competitive receptor occupancy of GnRH-r [337].

GnRH antagonists have been introduced clinically to prevent premature endogenous LH surge to replace the GnRH agonists that require time for a state of desensitization to be reached and, to start with, LH secretion actually increases (flare-up). With GnRH antagonists, immediate blockade of pituitary gonadotropins secretion when premature luteinization during IVF-ET stimulation is imminent seemed an obvious approach. Several studies of dose and treatment schedules have been done, [338,339] and two general approaches have emerged. The first is a single subcutaneous injection of a large dose on about the eighth day of stimulation with gonadotropins. The alternative is multiple (five or six daily) injections of a small dose from about day 6 of stimulation until the day that hCG for final oocyte maturation is given.

The next step in the development of GnRH antagonists use was to establish whether a GnRH antagonist is at least as effective as a GnRH agonist as the established reference medication testing the regimen of repeated antagonist injections [340,341] or the regimen of a single-dose regimen [342].

With repeated injections, treatment with gonadotropins was found to be shortened by 1–2 days with slightly fewer follicles at the time of hCG injection so the number of recovered oocytes tends to be lower. No significant difference was found with respect to percentages of metaphase II oocytes, fertilization rates, and number of good quality embryos [340,341].

Pregnancy rates were high in both groups in all four studies but in every one the rate was lower in the antagonist group. A meta-analysis of the five randomized trials, showed an overall significantly lower rate of pregnancy of 5%. This meta-analysis unfortunately included the study that compared a single-dose analogue regimen with different gonadotropins starting dose as an additional variable [342]. It has been suggested that the larger numbers of oocytes and embryos with agonists allow better selection [343], although the numbers of good quality embryos do not seem to be different. A direct adverse effect on the embryo cannot yet be ruled out but is not likely.

Antagonist blockade allows immediate reversal of pituitary gonadotropins secretion. This means that in IVF-ET ovulatory ripening can be triggered via endogenous gonadotropins by using a GnRH agonist [344] with the advantage of prevention of ovarian hyperstimulation syndrome, which is thought to result at least in part from the prolonged LH effect of hCG [345]. Finally, the use of GnRH antagonist blockade of premature luteinization can be used in IVF-ET with very low or without any hormonal stimulation, lower risk of overstimulation, and simplification of the procedure [346].

A possible disadvantage of an antagonist in IVF-ET is its narrow therapeutic range with the currently advised doses for repeated injections. Patient compliance needs to be high because there is a risk of premature LH secretion if an injection is missed [337].

Another disadvantage is the unpredictable timing of the start of the IVF-ET procedure, which begins with the administration of FSH on day 3 of the woman's cycle and so depends on how regular her menstruation is. This problem may be solved by pretreatment with an oral contraceptive [335].

##### 4.1.3 Use of adjuvant medication during ovarian stimulation

###### Insulin sensitizers

The independent effect of insulin resistance on infertility treatment in PCOS is not well defined. Regardless of body weight, insulin-resistant PCOS women need higher gonadotropins doses during ovarian stimulation, and insulin resistance is also associated with a risk of multifollicular development and high cancellation rate [347]. Hyperinsulinemic PCOS women are more likely to produce lower quality oocytes that exhibit low fertilization rates after IVF-ET in association with lower implantation rates [348].

Improving insulin resistance with exercise, low-calorie diet and insulin-lowering drugs such as metformin, troglitazone and acarbose decrease insulin levels, correct the endocrine abnormalities induced by obesity and insulin resistance which may improve the outcome of infertility treatment [349-351]. This leads to the assumption that co-treatment with insulin-lowering drugs or weight reduction before and during down-regulation and ovarian stimulation.

Some studies did not find deleterious of insulin resistance on ovarian stimulation [352]. Metformin treatment has been reported to increase the number of mature oocytes retrieved from women with PCOS undergoing gonadotropins-stimulated IVF-ET and ICSI. A low dose of metformin of 500 mg twice daily was started on day 1 of the cycle prior to leuprolide acetate suppression, and continued to the day of the pregnancy test. Metformin treatment significantly increased the number of mature oocytes, fertilization rates, and number of embryos produced, but did not alter the total number of oocytes or peak E_2 _levels [353].

Later, the same authors reported that metformin therapy improves ovarian stimulation and IVF-ET outcomes in coasted patients with clomiphene-resistant PCOS [354]. No further published reports to date have examined this issue.

###### Corticosteroids

Successful use of corticosteroids in treatment of anovulatory infertility has been reported [355,356]. Later corticosteroids were used as adjuvant therapy together with clomiphene citrate or gonadotropins in ovulation induction. The theoretical basis for this application has not been fully elucidated, but it has been postulated that corticosteroid treatment could improve ovulation through reduced influence of the adrenal androgens on follicular development [357]. There have been reports on improved ovulation and pregnancy rates with adjuvant corticosteroid treatment in anovulatory women with elevated androgens or androgens within the higher range [358,359], as well as in normoandrogenic women [360]. Adding dexamethasone to gonadotropins in ovulation induction in women with normal serum concentrations of gonadotropins, androgens, and prolactin did not give an improved outcome in other studies [361].

Corticosteroids have also been used as adjuvant therapy in IVF-ET treatment. In 1986, Kemeter and Feichtinger [362] reported a significantly better pregnancy rate in a group of women with various infertility causes, except cycle abnormalities, undergoing IVF-ET with adjuvant prednisolone treatment, as compared to a group of women without adjuvant prednisolone. They stated that prednisolone would improve follicle maturation and thereby improve the pregnancy rate. In contrast, others [363] did not see any beneficial effects of adjuvant dexamethasone in a group of women with serum DHEA-S >2.5 μg/ml [6510 nmol/l] undergoing IVF-ET treatment, neither did Bider [361], in a group of women with tubal factor infertility after addition of dexamethasone.

In the above-mentioned study by Lobo [357], decreased serum concentrations of testosterone, unbound testosterone, and DHEA-S were noted after dexamethasone and clomiphene administration. In women who ovulated, testosterone and unbound testosterone increased again when clomiphene was added despite the continuation of dexamethasone. Decreased serum concentrations of DHEA-S and testosterone after ovarian stimulation with clomiphene citrate and human menopausal gonadotropins (HMG) and adjuvant prednisolone have been reported [362]. The other studies did not report data on androgen concentrations after glucocorticoid treatment. The present prospective, randomized, placebo-controlled study was performed to find out if adrenal suppression with prednisolone during ovarian stimulation before IVF-ET in a group of women with PCOS resulted in any changes in serum and follicular-fluid androgens. Clinical outcome variables, such as embryo quality, implantation rate, and clinical pregnancy rate were also noted.

Fridstrom [364] have reported that adjuvant glucocorticoids in ovarian stimulation before IVF-ET did not decrease the concentrations of adrenal androgens in serum and follicular fluid in PCOS. However, whether there were beneficial effects on ovum quality or implantation rate could not conclusively be determined in their study due to the small number of patients.

The addition of corticosteroids to ovarian stimulation protocols for assisted reproduction has been suggested in cases of recurrent pregnancy loss [365].

###### Progesterone antagonists: Receptor antagonist onapristone and mifeprisotone

Krusche found that after ovarian stimulation, the PR antagonist onapristone retarded endometrial transformation in the rabbit model. The authors concluded that since ovarian stimulation, used in human IVF-ET therapy, is frequently reported to cause an advancement of post-ovulatory endometrial development, a therapeutic application of PR antagonists to slow down such advanced endometrial transformation was suggested. Eventually, this modulation of advanced endometrial development may improve implantation rates [366].

#### 4.2 Improving embryology techniques

Improving embryo techniques is a very important approach to improve the outcome of assisted reproduction treatment. This includes, improving culture conditions and extended culture till the blastocyst stage as well as improving the accuracy for selecting viable embryos for transfer. Another strategy employs pre-implantation genetic diagnosis (PGD). In addition there are other strategies to improve the embryo quality including oocyte donation and micromanipulation techniques (assisted hatching, and cytoplasmic and mitochondrial transfer) [367].

##### 4.2.1 Improving embryo culture condition, selection and extended embryo culture (blastocyst transfer)

Over the past decade there has been considerable interest in optimizing culture media for supporting human embryos including reducing glucose concentrations [368], adding amino acids [369] and supplementing media with growth factors [370]. However, there have been no large prospective randomized studies on the effects of culture media on outcome after IVF-ET, apart from one, which reported that although glucose-free medium improved embryo quality, pregnancy rates were not increased [371]. All these improvements as well as the growing data on the composition of human female reproductive tract fluids and from embryo physiology studies has led to the proposition that extended embryo culture should take place till the blastocyst stage in more than a single medium formulation [372]. To this end, sequential media have been developed, tested extensively on animal models and subsequently used clinically [373].

One approach to increasing pregnancy rates is to improve the selection of viable embryos. Embryos are selected on the basis of morphology and rate of development, and the fastest developing embryos of the best morphology are selected for transfer. Although there is some correlation between morphology and blastocyst formation [374] and implantation [375], selection on the basis of morphology and rate of development remains an unsatisfactory and imprecise method of selecting viable embryos. It is impossible to identify visually with certainty, which embryos at early cleavage stages will subsequently arrest. One approach to improve the selection is the extended culture of the embryos to the blastocyst stage and transfer them on day 5 or 6, allowing the identification of developing embryos and the transfer of blastocysts, which are synchronous with the endometrium development, rather than cleavage stage embryos. It had been hoped also that, during extended culture, the chromosomally abnormal embryos so common at early cleavage stages [376] would arrest and fail to complete preimplantation development. However, although few human blastocysts have total chromosomal abnormality, a large number of blastocysts have various proportions of abnormal cells and are mosaic [377].

However, although many clinics have experienced increased success with extended culture, others have reported no benefit. So, it would be unwise to suggest that blastocyst culture and transfer represents a panacea for all clinics and all patients. On the contrary, transferring early stage embryos (zygot intrafallopian transfer) may help in repeated implantation failure rather than blastocyst transfer. [378].

##### 4.2.2 Preimplantation genetic diagnosis

Preimplantation genetic diagnosis (PGD) allows the diagnosis of a genetic disorder in an embryo before its implantation in the uterus. PGD involves the removal of one or two blastomeres from an eight-cell stage embryo after IVF-ET, analysis of the blastomeres using fluorescent in situ-hybridization (FISH) or PCR, and identifying affected embryos. As only unaffected embryos are transferred back to the uterus after PGD, termination can be avoided. PGD is of benefit to couples at risk of passing on a genetic disease to their offspring as well as for couples with repeated unexplained IVF-ET failures. The transfer of unaffected embryos is believed to enhance the success rate of IVF-ET. Unfortunately, a large number of oocytes need to be retrieved for a successful PGD cycle, as not all will fertilize, cleave, undergo successful biopsy and be identified as suitable for transfer. Although most embryos (96%) survive biopsy, the pregnancy rates after transfer of biopsied embryos (16% per treatment cycle) [377] are less than those after conventional IVF-ET (23% per cycle) [379]. However, PGD is still in its early developmental stages [14].

##### 4.2.3 In-Vitro Maturation of Oocytes

Immature oocytes can be aspirated from small follicles of > 2 mm in diameter. Resumption of meiosis in fully-grown oocytes, germinal vesicle breakdown, extrusion of the first polar body and acquisition of the ability to be fertilized may all occur during in vitro maturation (IVM). Although the first human birth after IVM was achieved 20 years ago [380], a few cases have been reported subsequently, including births after the aspiration and IVM of oocytes retrieved from women who have natural or partially stimulated cycles [381] and women with PCOS [382]. When oocytes are removed from small antral follicles and placed in culture, approximately 60% will have undergone nuclear maturation within 48 h. After exposure to spermatozoa, about 40% of oocytes will undergo normal fertilization, exhibiting two pronuclei and extruding the second polar body. Between 20 and 25% of fertilized oocytes will undergo cleavage [383]. However, pregnancy rates after the transfer of such embryos have been extremely low (1–2%) and most embryos arrest between the four- and eight-cell stages with only few encouraging results [384]. Several factors may influence the fertilization, cleavage, blastocyst and pregnancy rates achieved after IVM, including the hormonal environment in vivo or in vitro and the composition of the culture medium [385]. Further research in these areas will lead to greater understanding of oocyte maturation and should result in improved implantation and pregnancy rates [14].

##### 4.2.4 Micromanipulation techniques

###### Intracytoplasmic sperm injection (ICSI)

The use of small numbers of spermatozoa during IVF-ET results in fertilization failure. In these cases, it is possible to inject a single spermatozoon into the ooplasm of a mature metaphase II oocyte, a procedure known as ICSI. The first successful pregnancies were in the early 1990s [10]. Since then, the technique has become a widely accepted treatment for couples with severe male factor infertility. Extending the indication of ICSI to include non-male factor infertility has been suggested to improve the fertilization and pregnancy rates. However, several studies have not supported the benefit of ICSI over conventional IVF-ET in improving the treatment outcome for non-male factor indications with growing consensus against applying ICSI for all cases undergoing IVF-ET [386-389].

###### Assisted hatching

To help embryos hatching from their zona pellucida during blastocyst expansion, different types of assisted hatching have been developed, including mechanical partial zona dissection or zona drilling, chemical zona drilling with acidic Tyrode's solution, and the laser technique [176]. However, there is controversy about the benefit of assisted hatching on the improvement of the implantation rate and pregnancy rate. Some investigators agreed as to the benefit of assisted hatching in women with advanced age, in women with repeated IVF-ET failure, and in the general population [177,178].

###### Mitochondrial injection and cytoplasm, and nuclear transfer

Van Blerkom suggested that the developmental competence of mouse and human early embryos is related to the metabolic capacity of the mitochondria [144]. Deletions and mutations in oocyte mtDNA may lead to mitochondrial dysfunction, influencing energy production and apoptosis in oocytes and early embryos, resulting in aberrant chromosomal segregation or developmental arrest [144]. In mice, cytoplasmic transfer from young to old oocytes improved older oocytes quality [390].

Cytoplasmic transfer from metaphase II donor oocytes to mature recipient oocytes from women with recurrent IVF-ET failure has been carried out in an attempt to restore normal growth in developmentally compromised oocytes and embryos [391].

Nuclear transfer has also been proposed for the treatment of mitochondrial disease [392] whereby a karyoblast containing metaphase II chromosomes from women with repeated failures of embryo development due to defective oocyte cytoplasm is fused to enucleated donor oocytes. The increased cost and complexity as well as the invasiveness of the procedure with the decreased implantation rates all make these procedures far from being applied routinely to improve the treatment outcome after assisted reproduction. Only selected cases might be expected to benefit from these newly developing techniques. It should be noted that currently in the United States, performance of cytoplasmic or nuclear transfer in humans can only be conducted following approval of the protocol by the FDA.

## PART TWO

### 1. Aromatase inhibition to improve outcome of treatment after ovarian stimulation

#### 1.1 Introduction

We hypothesize that aromatase inhibitors can be used to improve the treatment outcome after ovarian stimulation either alone, or in combination with IUI and assisted reproductive technology. The use of aromatase inhibitors during ovarian stimulation may have several benefits including: (1) The enhancement of implantation by lowering the supraphysiological levels of estrogen attained during ovarian hyperstimulation that is believed to adversely affect the development of the endometrium, oocytes and embryo, as well as other possible targets. (2) Reducing gonadotropins dose required for achievement of optimum ovarian stimulation. This would reduce possible deleterious direct effects of exogenous gonadotropins injection in addition to reducing the cost of treatment. (3) Other possible benefits such as improvement of ovarian response to FSH stimulation in poor responders, and prevention of premature endogenous gonadotropin surge, as well as lower risk of severe ovarian hyperstimulation syndrome.

The improvement in implantation, as well as the reduced cost of treatment by decreasing the gonadotropins dose required for ovarian hyperstimulation would encourage the policy of transferring one embryo to minimize the risk of multiple gestation. This would have a tremendous economic impact on the practice of assisted reproduction, as well as cost of health care for multiple gestation worldwide.

#### 1.2 Outline of the use of aromatase inhibitors for ovarian stimulation

In the following section, we present brief outline on the aromatase enzyme, estrogen biosynthesis and the development of aromatase inhibitors followed by a summary of the available data concerning the use of aromatase inhibitors for ovarian stimulation.

##### The aromatase enzyme

Aromatase is a microsomal member of the cytochrome P450 hemoprotein-containing enzyme complex superfamily (P450 arom, the product of the CYP19 gene) that catalyzes the rate-limiting step in the production of estrogens, that is, the conversion of androstenedione and testosterone via three hydroxylation steps to estrone and E_2 _respectively [393]. Aromatase activity is present in many tissues, such as ovaries, brain, adipose tissue, muscle, liver, breast tissue, and in malignant breast tumors. The main sources of circulating estrogens are the ovaries in premenopausal women and adipose tissue in postmenopausal women [394,395].

##### The development of aromatase inhibitors

Aromatase is a good target for selective inhibition because estrogen production is a terminal step in the biosynthetic sequence. Several aromatase inhibitors have been utilized in clinical studies over the last 20 years. The most successful, third generation aromatase inhibitors are licensed for breast cancer treatment [396]. Aromatase inhibitors have been classified in a number of different ways, including first-, second-, and third-generation; steroidal and non-steroidal; reversible (ionic binding), and irreversible (suicide inhibitor, covalent binding) [397]. Table 4 lists the different classes of aromatase inhibitors.

**Table 4:** Different classes of aromatase inhibitors:

Generation	Non-Steroidal	Steroidal
First Generation	Aminogltethimide	
Second Generation	Rogletimide	Formestane
	Fadrozole	
Third Generation	Anastrozole	Exemestane
	Letrozole	
	Vorozole	

**Table 5:** The different degrees of whole body aromatase inhibition by the various aromatase inhibitors

Aromatase inhibitor	Dose	Mean percentage of total body aromatase inhibition
Aminoglutethimide in conjunction with hydrocortisone	1000 mg plus 40 mg of hydrocortisone (daily)	90.6
Formestane	250 mg (every 2 weeks, intramuscularly)	84.8
Exemestane	25 mg/day	97.9
Fadrozole	2 mg/day	82.4
Anastrozole	1 mg/day	97.3
Letrozole	2.5 mg/day	>99.1
Vorozole	1 mg/day	93

Steroidal aromatase inhibitors are androstenedione analogues that act as a false substrate and bind irreversibly to the androgen-binding site of the enzyme [398]. Non-steroidal aromatase inhibitors exert their function through binding to the heme moiety of the cytochrome P450 enzyme [399]. The first of these inhibitors to be used clinically was aminoglutethimide, which induces a medical adrenalectomy by inhibiting many other enzymes involved in steroid biosynthesis [400]. Although aminoglutethimide is an effective hormonal agent in postmenopausal breast cancer, its use is complicated by the need for concurrent corticosteroid replacement, in addition to side effects like lethargy, rashes, nausea and fever that result in 8–15% of patients stopping treatment [401,402]. The lack of specificity and unfavorable toxicity profile of aminoglutethimide led to the search for more specific aromatase inhibitors. In addition, the above mentioned aromatase inhibitors were not able to completely inhibit aromatase activity in premenopausal patients.

##### Third generation aromatase inhibitors

The third-generation anti-aromatase agents commercially available include two non-steroidal preparations, anastrozole and letrozole, and a steroidal agent, exemestane. Anastrozole and letrozole are often referred to as aromatase inhibitors, whereas exemestane is called an aromatase inactivator [403,404]. Anastrozole, ZN 1033, (Arimidex^®^) and letrozole, CGS 20267, (Femara^®^) are selective aromatase inhibitors, available for clinical use in North America, Europe and other parts of the world for treatment of postmenopausal breast cancer. These triazole (antifungal) derivatives are reversible, competitive aromatase inhibitors, which are highly potent and selective. At doses of 1–5 mg/day, they inhibit estrogen levels by 97% to >99% resulting in estrogen concentrations below detection by most sensitive immunoassays. Table 5 shows the relative potencies of different aromatase inhibitors. Aromatase inhibitors are completely absorbed after oral administration, with mean terminal half-life (t_1/2_) of approximately 45 hours (range, 30–60 hours). They are cleared from the systemic circulation mainly by the liver. Gastrointestinal disturbances account for most of the adverse events, although these have seldom limited therapy. Other adverse effects are asthenia, hot flashes, headache, and back pain [406]. The average wholesale cost for a month's supply (thirty tablets) of aromatase inhibitors is about $150 to $250 [407]. Table 6 lists the clinical advantages of the third generation aromatase inhibitors as regards potential use for ovarian stimulation.

**Table 6:** Advantages of the third generation aromatase inhibitors:

• Extremely potent in inhibiting the aromatase enzyme
• Very specific in inhibiting the aromatase enzyme without significant inhibition of the other steroidogenesis enzymes
• Orally administered
• 100% bioavailability after oral administration
• Rapid clearance from the body (Short half-life, ~45 hours)
• No accumulation of the medications or their metabolites
• No significant active metabolites
• Well tolerated on daily administration for years
• Few side effects
• Very safe without significant contraindications
• Relatively inexpensive

##### Hypotheses behind the use of aromatase inhibitors for ovarian stimulation

In the late 1990s, we explored the hypothesis that it might be possible to mimic the action of clomiphene citrate, without depletion of ER, by administration of an aromatase inhibitor in the early part of the menstrual cycle. We hypothesized that the result of blocking estrogen production would be release of the hypothalamic/pituitary axis from estrogenic negative feedback, thereby increasing gonadotropin secretion and resulting in stimulation of ovarian follicular development. This first hypothesis is referred to as CENTRAL hypothesis. The selective non-steroidal aromatase inhibitors have a relatively short half-life (approximately 40 hours) compared to clomiphene citrate, and would be ideal for this purpose since they are eliminated from the body rapidly [408,409]. In addition, no adverse effects are expected on estrogen target tissues, since no ER down-regulation occurs as observed in clomiphene citrate treatment cycles.

We subsequently developed a second hypothesis, which was referred to as the PERIPHERAL hypothesis to explain another mechanism of action of the aromatase inhibitors in ovarian stimulation. We believe that aromatase inhibitors also act locally in the ovary to increase follicular sensitivity to FSH stimulation. This may result from the temporary accumulation of intraovarian androgens, since conversion of androgen substrate to estrogen is reversibly blocked by aromatase inhibition. There are data supporting a stimulatory role for androgens in early follicular growth in primates [410]. Testosterone was found to augment follicular FSH receptor expression in primates suggesting that androgens promote follicular growth and estrogen biosynthesis indirectly by amplifying FSH effects [411,412]. In addition, androgen accumulation in the follicle may stimulate insulin-like growth factor I (IGF-I), along with other endocrine and paracrine factors, which may synergize with FSH to promote folliculogenesis [413-416]. This hypothesis still waits for evidence to support it.

###### 1.2.1 Use of aromatase inhibitors alone for ovarian stimulation

In the last few years, we have worked on the development of aromatase inhibitors for ovarian stimulation and infertility management and reported interesting success in different applications as follows:

####### Induction of ovulation in anovulatory women

Based on our CENTRAL hypothesis of using an aromatase inhibitor for induction of ovulation (as explained above), the success of an aromatase inhibitor in inducing ovulation in patients with PCOS was reported [417-420].

####### Augmentation of ovulation in ovulatory women

After demonstrating success in inducing ovulation in anovulatory women, we proceeded to test whether aromatase inhibition might enhance the release of endogenous gonadotropins enough to stimulate the development of multiple follicles in ovulatory women and result in augmentation of ovulation or even controlled ovarian hyperstimulation. The use of an aromatase inhibitor for augmenting ovulation in patients with ovulatory infertility was successful in women with unexplained infertility, endometriosis, and women undergoing therapeutic donor insemination, and in ovulating partners of infertile men [419,420].

####### Induction and augmentation of ovulation after clomiphene citrate failure

For over 40 years, clomiphene citrate has been the most commonly used treatment for the induction and augmentation of ovulation, accounting for about two thirds of the fertility drugs prescribed in the United States [421,422]. In spite of the high ovulation rate associated with the use of clomiphene citrate, the pregnancy rate is much lower than anticipated. This is particularly true when considering pregnancy rate per cycle after three cycles of CC treatment [421], with higher than expected incidence of miscarriage in conception cycles [255]. Such discrepancy is believed, as explained earlier, to be due to the peripheral antiestrogenic effect of clomiphene citrate particularly at the level of the cervical mucus and endometrium, and which manifest themselves even in the presence of gonadotropin treatment superovulation. The accumulation in the body of the isomers of clomiphene citrate due to the long half-life (several days to weeks) adds to the persistence of the antiestrogenic effect [261,262].

In order to improve the outcome of clomiphene citrate treatment, various approaches have been suggested to overcome the antiestrogenic effect including concomitant estrogen administration. Some investigators reported increased endometrial thickness and improved pregnancy rates with this approach [423-425] while others have reported no benefit [426] or even a deleterious effect of estrogen administration [427]. Another approach was to administer clomiphene citrate earlier during the menstrual cycle [428], to allow the anti-estrogenic effect to wear off earlier before the critical period of fertilization and implantation. A third approach has been to combine another selective ER modulator like tamoxifen, which has more estrogen agonistic effect on the endometrium with clomiphene citrate [429] or to use it as an alternative to clomiphene citrate [430]. However, none of these strategies have proved to be completely successful in avoiding the peripheral antiestrogenic effects of clomiphene citrate. In addition to a discrepancy between ovulation and pregnancy rates with clomiphene citrate treatment, 20% to 25% of anovulatory women are resistant to clomiphene citrate and fail to ovulate at doses up to 200 mg daily.

The success of aromatase inhibition in inducing and augmenting ovulation encouraged trying aromatase inhibitors in cases of clomiphene citrate failure that was found to be successful in achieving ovulation and pregnancy [419,420]. As explained above, the significantly shorter half-life of the third generation aromatase inhibitors compared to clomiphene citrate, allows rapid elimination from the body [408,409]. In addition, since no ER down-regulation occurs, any adverse effects on estrogen target tissues, as observed in clomiphene citrate treated cycles, is expected.

###### 1.2.2 Adjunct use of aromatase inhibitors for ovarian stimulation

We investigated the idea of combining an aromatase inhibitor with FSH injections to reduce the dose of FSH required to achieve optimum controlled ovarian hyperstimulation, without adverse antiestrogenic effects [431,432]. A significant reduction in the FSH dose required (from 45% to 55% in women with PCOS and unexplained infertility) has been reported [433] without evidence of peripheral antiestrogenic effects [432,433].

####### Improving ovarian response to FSH stimulation in poor responders

Reducing FSH dose required for optimum controlled ovarian hyperstimulation encouraged us to explore the use of an aromatase inhibitor in conjunction with FSH to improve response to ovarian stimulation in poor responders. In a selected group of women who had a poor response to FSH stimulation in at least two prior treatment cycles, adding an aromatase inhibitor resulted in improvement in the response to FSH stimulation. All patients developed a significantly greater number of mature ovarian follicles and almost a third of the treatment cycles resulted in pregnancy. In addition, the dose of FSH required to achieve such optimum response was significantly less than the dose used in the prior cycles in which FSH was used alone [434,435].

####### Developing alternative regimens for administering aromatase inhibitors for ovarian stimulation

During all the above-mentioned studies, the aromatase inhibitor, letrozole, was administered orally as a daily dose of 2.5 mg from day 3 to day 7 of the menstrual cycle. Based on the pharmacokinetics of the new aromatase inhibitors (almost 100% bioavailability after oral administration, ~2 days half-life and no accumulation or significant metabolite accumulation), we thought of a potentially more convenient method of administering an aromatase inhibitor for ovulation induction. We hypothesized that significantly higher concentration of the aromatase inhibitor can be achieved early in the menstrual cycle with faster clearance later in the menstrual cycle with the administration of a high single dose of the aromatase inhibitor early in the menstrual cycle such as on day 3. A single dose regimen would satisfy two goals: first, achieving maximum estrogen suppression early in the menstrual cycle when it is desired, and second, to allow clearance of the aromatase inhibitor before the critical final stage of fertilization and embryogenesis, to maximize safety and avoid any possible undesirable effects of the aromatase inhibitors. Single dose administration of an aromatase inhibitor was found successful in inducing ovulation with ovulation and pregnancy rates comparable to the 5-day regimen [436].

####### Limitations of preliminary data on use of aromatase inhibitors for ovarian stimulation

In all our clinical trials described above, results were not obtained from randomized, prospective, placebo controlled studies, the optimum research design. However, due to the experimental nature of the use of the aromatase inhibitors for ovarian stimulation, which to our knowledge, has never been used before, we believed that the present observational trials were mandatory before proceeding into any definitive randomized studies.

The encouraging results of our data have led other investigators from different centers world-wide to study the use of aromatase inhibitors for ovarian stimulation and in general have reported findings similar to ours in terms of the success of aromatase inhibitors in infertility treatment [437-450].

In a randomized, prospective study, a superior uterine environment has been found in patients treated with an aromatase inhibitor compared with clomiphene citrate, reflecting the lack of the antiestrogenic effects with aromatase inhibitor treatment. Although a non-significant increase in pregnancy was observed in patients who received aromatase inhibitor treatment (16.7 versus 5.6% per patient, P = 0.55), this almost three-fold increase in pregnancy rate would have required about ten more patients per group to reach statistical significance. The superiority of a single dose administration of an aromatase inhibitor was also reported when compared to clomiphene citrate [439]. In other studies, the use of an aromatase inhibitor in conjunction with FSH was found to reduce the FSH dose [440] and improve response to ovarian stimulation in poor responders [441,442], confirming our previous reports.

In another prospective randomized trial, Biljan [438] studied two doses (2.5 and 5 mg per day) of the aromatase inhibitor, letrozole for ovarian stimulation in patients with unexplained infertility. They found patients treated with a higher dose of letrozole developed more follicles without a detrimental effect on endometrial development. The potential for different doses and regimens of administration of aromatase inhibitors for ovarian stimulation still requires a lot of study, but the use of aromatase inhibitors at high doses should be cautiously considered.

In all reports and most of studies of other investigators, the aromatase inhibitor, letrozole was the one used. However, anastrozole, another third generation aromatase inhibitor similar to letrozole, was used in other studies [443,444]. Due to the similar pharmacokinetics and pharmacodynamics, including similar potencies and specificity in inhibiting the aromatase enzyme, we believe there is likely to be no difference between the third generation aromatase inhibitors in their efficacy for ovarian stimulation. It does, however, need to be determined what dose of anastrozole is required to be equivalent to letrozole.

######## Future avenues for the use of aromatase inhibitors for infertility management

It seems that there are many interesting areas of research that need exploration as regards the development of aromatase inhibitors for infertility management. These directions for research would include: confirming the available preliminary data on the success of aromatase inhibition in induction and augmentation of ovulation, as well as reducing the dose of FSH needed for ovarian stimulation, improving response in poor responders, and finding the optimum regimen for administering aromatase inhibitors for infertility treatment

Moreover, the use of aromatase inhibitors for new applications including in-vitro maturation and prevention of severe ovarian hyperstimulation syndrome and endometriosis-related infertility are interesting future avenues for aromatase inhibitors potential use in infertility management.

In addition the use of aromatase inhibitors to improve the outcome of treatment after assisted reproduction as discussed earlier in this review is an exciting area of application.

#### 1.3 Use of aromatase inhibitors to improve treatment outcome after ovarian stimulation and assisted reproduction

In addition to using aromatase inhibitors, ALONE, for ovarian stimulation, their use during assisted reproduction carries several potentials to improve the treatment outcome.

##### Improving implantation rates

We hypothesize that aromatase inhibition during assisted reproduction may improve the implantation rate mainly by reducing the estrogen levels attained during COH. In addition, two other mechanisms may apply including reducing the dose of FSH required for optimum COH as well as applying much simpler stimulation protocols that do not require the use of GnRH analogues, hence avoiding any possible direct deleterious effects of FSH and GnRH analogues on the endometrium.

###### 1.3.1 Lowering supraphysiological estrogen levels

As discussed above, the undesirable effects of ovarian stimulation on the outcome of infertility treatment are believed to be due to the supraphysiological levels of estrogen irrespective to whatever mechanisms explain for that (various postulated mechanisms were discussed above). So, lowering estrogen levels may be associated with improved outcome of treatment in terms of improving the implantation and pregnancy rates in addition to lowering risk of sever ovarian hyperstimulation syndrome.

Reducing estrogen synthesis by aromatase inhibition seems to be an exciting idea to ameliorate the deleterious effects of the supraphysiological levels of estrogen on the endometrium, the developing oocyte and embryo as well as the luteal.

Until recently there was no suitable aromatase inhibitor that could be used clinically to reduce estrogen levels during ovarian stimulation. This is because the available aromatase inhibitors were not safe for clinical application during ovarian stimulation due to lack of specificity in inhibiting the aromatase enzyme without inhibiting other steroidogenesis enzymes (e.g. aminoglutethimide). The other aromatase inhibitors (steroidal androstenedione analogues) were irreversible in their effect on the aromatase enzyme besides being parentrally administered. Most important, these old aromatase inhibitors were not potent enough to inhibit the aromatase enzyme and lower estrogen levels in women of the reproductive age group. However, the third generation non-steroidal aromatase inhibitors group is very potent and specific in inhibiting the aromatase enzyme reversibly. These new aromatase inhibitors are orally administered with very high safety profile and well tolerability. Moreover, they are cheap with a relatively short half-life [~45 hours], and already approved for clinical use to reduce estrogen production in postmenopausal women with breast cancer.

They have not been used in women of the reproductive age group. However, we have found these aromatase inhibitors to be effective in inhibiting the aromatase enzyme and effectively decrease estrogen levels in women of the reproductive age group during their successful use for ovarian stimulation [417,418,431,432].

In our experience with the use of an aromatase inhibitor for ovarian stimulation, estrogen levels were significantly lower (especially E_2 _level/mature follicle) when compared with conventional stimulation protocols (clomiphene citrate, FSH and clomiphene citrate plus FSH). Such low E_2 _levels may be beneficial and explains at least partially the improved outcome of treatment in terms of achieving promising high pregnancy rates during aromatase inhibitor treatment.

It may not be only the supraphysiological estrogen levels, which explain totally for the reduced implantation rate during ovarian stimulation cycles. Local paracrine factors, or possibly other undetermined factors may be responsible for the reduced implantation rate during COH. In this case the aromatase inhibitors may not offer a complete solution to overcome such drawbacks of ovarian stimulation. However, the idea is exciting and seems to be promising and warrant trials because high estrogen levels may explain, at least in part, for the deleterious effects of ovarian stimulation on treatment outcome.

In ddition, we think that, reducing estrogen levels achieved during induction of ovulation by using aromatase inhibitors may prevent the significant increase in leptin concentrations avoiding its possible deleterious effects on the outcome of treatment as explained above. Because elucidation of leptin's specific role in reproductive function has been challenging, with conflicting results reported by various investigators, it is still quite early and highly speculative to hypothesize a link between aromatase inhibitors use and the role of leptin during induction of ovulation. However, the available strong data about a possible role of leptin in mediating reproductive disorders especially in obese women and the firm findings of a regulatory effect of estrogen on leptin production make this hypothesis exciting and interesting enough to warrant future investigation.

###### 1.3.2 Reducing gonadotropins requirements for optimum ovarian stimulation

As mentioned earlier, the use of an aromatase inhibitor significantly reduces the dose of FSH required for optimum COH. In addition to the significant economic benefit, we believe that reducing the dose of FSH may improve the treatment outcome of ovarian stimulation by reducing any possible deleterious effect of the exogenously administered FSH on the endometrium, developing oocyte or other targets.

####### Other possible benefits

It is believed that the endogenous LH surge arises once serum estrogen levels surpass a set threshold for a certain period of time [451-453].

The supraphysiological estrogen levels attained during ovarian stimulation are believed to cause premature release of the endogenous LH surge resulting in cycle cancellation during assisted reproduction. For that reason, GnRH analogues have been the standard of practice during most of the stimulation protocols for assisted reproduction to prevent the occurrence of endogenous LH surge either by direct inhibition of LH surge (GnRH antagonists) or by down-regulation of the GnRH receptors at the pituitary levels resulting in pituitary desensitization and prevention of the release of endogenous LH [454,455].

Unfortunately, the use of GnRH analogues (which is a crucial part of the stimulation protocol to prevent cycle cancellation as explained above), is associated with several problems including increasing the dose of FSH required to achieve optimum COH (due to suppressing the endogenous gonadotropin secretion, and possible peripheral effect at the level of the ovaries), as well as the luteal phase defect due to a dysfunctional corpus luteum function secondary to persistent endogenous LH suppression as explained earlier. In addition, there is rising evidence of a possible direct deleterious effect of the GnRH analogues, especially the antagonist at the level of the endometrium [456].

The use of an aromatase inhibitor to reduce estrogen levels attained during COH may be effective in preventing the occurrence of premature ovarian surge. This would avoid the use of GnRH analogues during stimulation protocols for assisted reproduction which has several advantages including prevention of the possible deleterious effects of these agents as mentioned above in addition to reducing the cost of treatment as well allowing implementing much simpler stimulation protocols during assisted reproduction.

####### Aromatase inhibitors for endometriosis-related infertility

The expression of aromatase enzyme in endometriotic tissues with the significant role of locally produced estrogen in endometriosis progression [457] suggests a benefit of aromatase inhibitors in endometriosis-related infertility. The inhibition of local estrogen production in endometrial implants, and the lower peripheral estrogen levels associated with the use of aromatase inhibition for ovulation induction, is expected to protect, to some degree, against progression of endometriosis during infertility treatment.

#### 1.4 Concerns regarding the use of aromatase inhibitors in infertility treatment

There are three main concerns that may arise regarding the use of the aromatase inhibitors to improve the outcome of treatment after ovarian stimulation and assisted reproduction. They include the possible deleterious effect of the low follicular estrogen milieu on the development of the oocytes, the possible direct effect of the aromatase inhibitors on the oocyte development, fertilization or embryogenesis, and the accumulation of the androgens that may result from inhibition of their conversion into estrogens.

##### 1.4.1 Effect of low estrogen milieu on the developing oocyte

Palter et al. have reviewed the question whether an estrogen-free (or at least very low estrogen) intrafollicular environment is compatible with follicular development, ovulation, and corpus luteum formation [458]. The authors concluded that markedly reduced to nonexistent intrafollicular and circulating concentrations of estrogen are compatible with follicular "expansion", retrievable and fertilizable oocytes, as well as with cleavable and apparently transferable embryos. The authors drew their conclusion after discussing lessons from data in the literature including cases of deficiency of the **17**-hydroxylase/17–20 lyase [459-466] and 3β-hydroxysteroid dehydrogenase [467,468] and the aromatase enzyme [469-473] as well as cases of severe hypogonadotropism [474-478]. In addition, data on the use of aromatase inhibitors in animals were reviewed [479-482].

###### Cases of enzyme deficiency leading to a status of very low estrogen levels

####### 17 alpha-hydroxylase/17–20 lyase deficiency

17alpha-hydroxylase/17–20 lyase deficiency is one form of congenital adrenal hyperplasia, which is associated with marked impairment of glucocorticoid, androgen, and estrogen biosynthesis [459]. Women suffering from this enzyme deficiency suffer from hypergonadotropic hypogonadism and sexual infantilism. However, early reports noted the presence of many primary and secondary follicles in ovarian material [460] and many of them had bilateral multicystic ovaries at the time of laparotomy [461-463].

It is interesting that Rabinovici reported on a patient afflicted with virtually complete 17-hydroxylase/17–20 lyase deficiency that, despite castrate levels of estrogens, underwent an apparently successful induction of ovulation associated with progressive follicular expansion and oocyte retrieval, IVF-ET, and early embryonic cleavage followed suit [464].

####### 3β-hydroxysteroid dehydrogenase [3β-HSD] deficiency

The deficiency of 3β-HSD is associated with markedly reduced levels of estrogen. However, the existence of normal ovulatory function in a woman with late-onset of a mild form of 3β-HSD has been reported [483].

In a group of cycling female Rhesus monkeys exposed to ovulation induction with hFSH and hFSH + hLH in the absence or presence of the 3β-HSD inhibitor trilostane given on days 1–8 of the menstrual cycle [467], apparently healthy oocytes were obtained by follicular aspiration 34 h after hCG administration. Importantly, treatment with the 3β-HSD inhibitor, trilostane, led to a reduction in serum E_2 _levels to 7% of that of control animals throughout the follicular phase. Despite this dramatic reduction in E_2 _levels, neither the total number of large antral follicles per animal (17 ± 1 vs. 18 ± 2) nor their size distribution differed significantly from 3β-HSD inhibitor-untreated controls. Furthermore, treatment with the 3β-HSD inhibitor did not alter the overall maturation pattern of collected oocytes (atretic, prophase I, metaphase I, or metaphase II). However, the authors found a reduction in the percentage of metaphase II oocytes that were successfully fertilized (15 vs. 65%). Moreover, metaphase oocytes that required more than 8 h to complete meiosis in vitro failed to fertilize in three of four animals receiving 3β-HSD inhibitor relative to controls (31%). These observations suggest that follicular development and the completion of meiosis may be unaffected by the low estrogen levels but that cytoplasmic oocyte maturation and/or function could be unfavorably affected [467].

####### Aromatase enzyme deficiency and the use of aromatase inhibitors

Obviously, aromatase (estrogen synthase) enzyme deficiency is associated with marked decrease or almost absence of estrogen production.

Extreme examples of complete aromatase deficiency due to mutations in the aromatase gene, CYP19 gene, in adult human females, however, were reported [469,470]. The affected patients suffered from ambiguous external genitalia, primary amenorrhea, sexual infantilism, and multicystic ovaries.

Morishima [471] reported on the aromatase deficiency in a 28-yr-old 46 XX proband followed since infancy. Null mutant mice for aromatase gene [ArKO] were generated [484], thereby affording the opportunity to examine the role of estrogen in the follicular development in the mouse ovary. Evaluation of the ovaries revealed the presence of many large follicles filled with granulosa cells and evidence of antrum formation, but no corpora lutea. The ovarian phenotype degenerated with age upon the appearance of hemorrhagic cystic follicles and the loss of secondary and antral follicles coincident with the infiltration of macrophages and with stromal hyperplasia [485-488]. Therefore, the ArKO females are infertile, due primarily to a complete lack of ovulation.

However, recently, Jones et al., reported that oocytes were harvested from the ovaries of 4- and 7-week old ArKO, wild type and heterozygote mice stimulated with 5 IU PMSG. The number of immature oocytes harvested from ArKO females did not differ from the number collected from wild type or heterozygote littermates of either age group. Oocyte in vitro maturation rates also did not differ between the three genotypes or two age groups, with almost 75% of the immature oocytes progressing to metaphase II. Chromatin staining confirmed the arrest of these oocytes at the second meiotic division with chromatin staining clearly present in the oocyte and polar body. Mature oocytes were inseminated and IVF-ET rates did not differ between the three genotypes or two age groups, with fertilization occurring in approximately 67% of oocytes. Fertilized oocytes were cultured to blastocysts. Again, blastocyst development rates did not differ between the groups, with approximately 65% of the zygotes developing into blastocysts. Blastocyst morphology was similar across all of the groups [489].

These results indicate that ArKO oocytes are competent to develop to at least the implantation stage. The authors concluded that estrogen might not be required for the production and maturation of developmentally competent oocytes. Rather, its role in folliculogenesis is probably via regulation of the hypophysial pituitary gonadal axis and thus gonadotropin secretion [489].

Regarding the effect of an estrogen-free/poor intrafollicular environment on gametogenic maturation, Palter et al. [458] concluded that the effect is a negative one. Their conclusion was based on a number of primate studies, which indicated that an estrogen-free/poor intrafollicular environment is associated with marked decrements in the rates of meiotic maturation and fertilization [467,490]. In addition data derived from rodent models suggested that further suggested a compromise in early embryonic development [491].

In cycling female rhesus monkeys, the aromatase inhibitor, 1,4,6-androstatrien-3, 17-dione (ATD) was used to inhibit estrogen production during gonadotropin ovarian stimulation [479]. Animals treated with ATD displayed a drastic reduction in serum 17β-E_2 _levels to 37% of that of controls within 8 h of ATD treatment and to 16% of control by the day of hCG injection. In turn, the circulating levels of androstenedione rose. Despite the drastic reduction in the circulating levels of E_2 _and the increase in the circulating levels of androgens, the overall number of large antral follicles [16 ± 3 for controls and 20 ± 3 for ATD-treated] and their size distribution (as assessed by ultrasonography) proved comparable for control and ATD-treated animals. Similarly, no difference was noted in the number of oocytes collected or in the proportion of oocytes reinitiating meiosis (MI at the time of collection). In contrast, ATD-treated animals displayed a marked increase (31 vs. 11%) in the proportion of prophase I oocytes. Moreover, ATD-treated oocytes displayed retarded in vivo completion of maturation to MII (4% vs. 26%). Interestingly, the latter retardation was not observed in vitro. Furthermore, two of the four ATD-treated animals yielded oocytes that were morphologically abnormal. Finally, oocytes from ATD-treated animals displayed significantly reduced rates of fertilization (9% vs. 25%) as compared with controls. However, the cleavage rate after successful fertilization was similar for ATD-treated vs. ATD-untreated controls. In vitro cultures of granulosa cells collected at the time of oocyte aspiration revealed equivalent 24-h progesterone production in treated and control animals. The authors concluded that these findings suggest that the acute reduction in E_2 _levels during the terminal stage of gonadotropin stimulation had little effect upon follicular recruitment and expansion but an apparent detrimental effect upon gametogenic function may, in fact, exist. However, we cannot extrapolate from this study that the findings are simply due to the acute reduction of estrogen production as it is important to realize that there was a concomitant significant rise in androstenedione. Moreover, the time of administering an aromatase inhibitor during the latter part of the follicular phase as well as the irreversible nature of aromatase inhibition constitute important differences from our model of using a REVERSIBLE aromatase inhibitor, TEMPORARILY, EARLY in the menstrual cycle that has a SHORT half-life.

Selvaraj [480,481] and Shetty [482] examine the effects of blocking estrogen biosynthesis during the follicular phase on follicular maturation in the adult female bonnet monkey. In their studies, they used one of the third-generation REVERSIBLE, aromatase inhibitors (CGS 16949A) starting EARLY on day 3 of the menstrual cycle. There were 53% and 70% reductions in the basal and surge levels of E_2_, respectively without obvious effect on follicular maturation, ovulation, and luteal function as assessed by serum hormone profiles as well as by laparotomy. Moreover, the concurrent administration of FSH and an aromatase inhibitor resulted in the suppression of the FSH-induced increase in the circulating levels of E_2 _(by 100%) with no effect noted on either the number of follicles developed or their size relative to control. In addition, granulosa and theca cells, removed on day 9 of the treatment cycle, were responsive to gonadotropins in vitro, disclosing no evidence of a deleterious effect on the cellular development and maturation of follicular cells.

Terry studied a possible role of high E_2 _levels in mediating the adverse effects of hyperstimulation with PMSG on early embryonic development in the rat [491]. They used the aromatase inhibitor, 4-hydroxyandrostenedione (4-OHA), to inhibit endogenous E_2 _production. The authors conducted three experiments. In the first, varying doses of 4-OHA were administered either concurrently with hCG to pro-estrus female rats hyperstimulated at early diestrus stage with 20 IU PMSG or alone into non-hyperstimulated pro-oestrus females. At high doses of 1000, 2000, or 5000 mg/rat, 4-OHA substantially improved the survival of embryos in hyperstimulated females with optimum protection at 2000 mg, while low doses of 100 and 500 mg/rat were ineffective. When administered alone, only the highest dose of 5000 mg/rat 4-OHA increased embryo count. In the second experiment, higher doses of PMSG were studied (30 or 40 IU), with or without 5000 mg/rat 4-OHA given at the time of hCG injection. PMSG proved to be more detrimental with increasing dose, and high doses of 4-OHA (5000 mg/rat) was needed to rescue embryos from death in the 30, but not 40, PMSG group. In the third experiment, the influence of the timing of 4-OHA treatment on its ability to improve the embryo count in hyperstimulated females was examined by introducing 4-OHA 24 h earlier, rather than at the time of hCG treatment. The results showed the importance of timing of 4-OHA administration, as 5000 mg/rat 4-OHA was able to restore embryo survival in the 40 PMSG hyperstimulated group only when it was administered 24 h before hCG injection. These results highlighted that 4-OHA, when administered at the appropriate time and dose, could reverse the negative effects of hyperstimulation from PMSG on early embryonic development. The authors concluded that this might be due to the suppression of estrogen production, thereby alleviating the supraphysiological level of E_2_, which is typically present in PMSG-treated females, which supports the hypothesis that excessive E_2 _is responsible for the negative effects of hyperstimulation with PMSG on early embryonic development.

The high success rates of ovulation and achievement of pregnancy in our reports on the use of aromatase inhibitor in ovarian stimulation, despite significantly lower estrogen levels could be due to several reasons. First: We used one of the third-generation "REVERSIBLE" aromatase inhibitors. Second: The aromatase inhibitor was administered EARLY in the follicular phase and for a limited period of time, which would allow the rapid clearance of the aromatase inhibitor from the body due to its SHORT half-life. Third: the reversible nature of the aromatase enzyme inhibition and the rising levels of FSH, which induce the expression of the aromatase enzyme, both would not allow for the estrogen levels to drop drastically below the low physiologic range. We believe that these low estrogen levels attained during the use of aromatase inhibitors for ovarian stimulation are compatible with healthy development of the oocytes, fertilization, embryo development, implantation and achievement of pregnancy. Fourth: the absence of significant rise in androgen levels would have prevented any possible unwanted effects on the terminal part of the oocyte maturation and ovulation.

##### 1.4.2 Direct effect of the aromatase inhibitors on the developing oocyte, fertilization and embryogenesis

As explained above, the short half-life of the aromatase inhibitors and limiting the administration to the early part of the follicular phase would allow the rapid clearance of the medications from the body before the important stage of fertilization and embryogenesis. This in addition to the absence of accumulation of the aromatase inhibitors of any of their metabolites would make them safe for the ovarian stimulation. We have reported a preliminary data on the pregnancy outcome after the use of aromatase inhibitors for ovarian stimulation supporting the safety of using these medications for such indication [492].

Data have been accumulating regarding the absence of deleterious effects in association with aromatase inhibitor treatment on follicle/oocyte maturation and embryo development in mice [492,493]. Treatment with the aromatase inhibitor, anastrozole was associated with similar number of total follicles found per animal (30.4 follicles/animal in the control group and 27 follicles/animal in the anastrozole-treated group) as well as comparable rates of development of embryos, morulae, blastocysts, and hatching blastocysts between the two groups (P = 0.20, 0.10, 0.44, and 0.38, respectively) [493].

##### 1.4.3 Effect of the accumulated androgens

As mentioned before, the rapid clearance of the aromatase inhibitors from the body due to their short half-life and the reversible nature of the aromatase enzyme inhibition as well as the rising levels of FSH, which induce the expression of the aromatase enzyme, all would not allow the clearance of any accumulating androgens by converting them to estrogen. The conversion of the androgens into estrogens (the step catalyzed by the aromatase enzyme) is a terminal step in the cascade of steroidogenesis. So, substrate (androgens) accumulation is not expected to be very significant, as other alternative earlier steps in the steroidogenesis pathways will work to clear out the accumulating androgens. For these reasons, pharmacokinetic studies on the new aromatase inhibitors reported the absence of significant androgen elevations or abnormal changes in other steroids in patients receiving aromatase inhibitors for breast cancer. In a subset of our patients who received an aromatase inhibitor for ovarian stimulation, we did not find significant change in the androgen levels while receiving the medication when compared to stimulation with gonadotropins alone (unpublished data).

Most recently we published data on the favorable pregnancy outcome after the use of letrozole for ovarian stimulation alone or in conjunction with gonadotropins [494]. This study looked at outcome of pregnancies achieved after ovarian stimulation with the aromatase inhibitor, letrozole, alone or in conjunction with gonadotropins. The study included a cohort of 394 pregnancy cycles achieved after letrozole and other ovarian stimulation treatments in a ddition to a control group of pregnancies spontaneously conceived without ovarian stimulation. These 394 pregnancy cycles were achieved as follows: 63 pregnancies with 2.5 mg of letrozole alone or with gonadotropins, 70 pregnancies with 5.0 mg of letrozole, 113 pregnancies with clomiphene alone or with gonadotropins, 110 pregnancies with gonadotropins alone, and 38 pregnancies achieved without ovarian stimulation. The study found pregnancies conceived after letrozole treatments were associated with similar miscarriage and ectopic pregnancy rates compared with all other groups. In addition, letrozole use was associated with a significantly lower rate of multiple gestation compared with clomiphene citrate. The findings of the stuy support the favorable pregnancy outcome and low multiple gestation rate in association with the use of aromatase inhibitors for ovarian stimulation [494].

### 5 Summary and conclusion

Ovarian stimulation particularly in conjunction with assisted reproductive technologies aims at stimulating the recruitment of several mature ovarian follicles that would enhance the chance of successful treatment by obtaining several embryos readily available for transfer into the uterus. It is obvious that supraphysiological levels of estrogen are inevitably attained during ovarian stimulation due to the significant contribution of estrogen production from each one of the several mature ovarian follicles. There is growing evidence that such supraphysiological estrogen levels are deleterious on the development of the endometrium, oocytes, embryos as well as other targets through different mechanisms. This is believed to explain, at least in part, the low pregnancy rates associated with assisted reproduction, mainly as a result of the persistently low implantation rates that have not improved impressively along almost three decades of assisted reproduction experience. This led several investigators to adopt the concept of minimal ovarian stimulation for assisted reproduction as an alternative approach to reduce the deleterious effects of the supraphysiological estrogen levels attained during routine controlled ovarian hyperstimulation. Unfortunately such approach, despite being logical, is associated with the major drawback of achieving fewer oocytes than desired, which, expectedly, reduces the chances of treatment success. Recently, we reported the success of the new generation aromatase inhibitors in ovarian stimulation. We found these agents to be effective in reducing the amount of estrogen production by mature ovarian follicles significantly. Furthermore, we found the use of these agents to be associated with improved ovarian response to stimulation by gonadotropins, resulting in significant reduction in the dose of gonadotropins required for controlled ovarian hyperstimulation. The novel idea of using aromatase inhibitors during ovarian stimulation for assisted reproduction combines the benefit of a significant reduction in estrogen production as well as gonadotropins dose, with the exiting advantage of achieving a good number of mature oocytes as a result of enhanced ovarian response to gonadotropins. This is expected to improve the various aspects of treatment outcomes after assisted reproduction including increased safety, reduced cost, as well as enhanced implantation rate. If confirmed in well-designed clinical trials, this would facilitate acceptance of the concept of single embryo transfer by infertile couples and practitioners, thereby reducing the epidemic of multiple births after assisted reproduction with its significant deleterious health and economical effects. This is particularly true in the light of recent data supporting the success of the approach of single embryo transfer [495-497].

## Authors' contributions

MFMM has written the review article, RFC and MPD have reviewed the manuscript
